# Dysfunction of the ubiquitin ligase E3A Ube3A/E6-AP contributes to synaptic pathology in Alzheimer’s disease

**DOI:** 10.1038/s42003-019-0350-5

**Published:** 2019-03-22

**Authors:** Markel Olabarria, Silvia Pasini, Carlo Corona, Pablo Robador, Cheng Song, Hardik Patel, Roger Lefort

**Affiliations:** 1 Taub Institute for Research on Alzheimer’s Disease & the Aging Brain and the Department of Pathology & Cell Biology, Columbia University, New York, NY 10032 USA; 2 Present Address: Department of Ophthalmology and Visual Sciences, Vanderbilt University Medical Center, Nashville, TN 37205 USA

## Abstract

Synaptic dysfunction and synapse loss are prominent features in Alzheimer’s disease. Members of the Rho-family of guanosine triphosphatases, specifically RhoA, and the synaptic protein Arc are implicated in these pathogenic processes. They share a common regulatory molecule, the E3 ligase Ube3A/E6-AP. Here, we show that Ube3A is reduced in an Alzheimer’s disease mouse model, Tg2576 mouse, which overexpresses human APP695 carrying the Swedish mutation, and accumulates Aβ in the brain. Depletion of Ube3A precedes the age-dependent behavioral deficits and loss of dendritic spines in these mice, and results from a decrease in solubility following phosphorylation by c-Abl, after Aβ exposure. Loss of Ube3A triggers the accumulation of Arc and Ephexin-5, driving internalization of GluR1, and activation of RhoA, respectively, culminating in pruning of synapses, which is blocked by restoring Ube3A. Taken together, our results place Ube3A as a critical player in Alzheimer’s disease pathogenesis, and as a potential therapeutic target.

## Abstract (web summary)

Markel Olabarria et al. show that the loss of Ube3A plays a critical role in synaptic dysfunction, using an Alzheimer’s disease mouse model. This study establishes a molecular pathway that maintains a synaptic function and suggests Ube3A as a potential therapeutic target for Alzheimer’s disease.

## Introduction

Alzheimer’s disease (AD), while classified as a neurodegenerative disease, is, at its core, a disease of synapses^1^. Mounting evidence suggests that impairment of cognitive abilities typically seen in the earliest clinical phases are due to prominent synaptic alterations and synapse loss, particularly in the entorhinal cortex (EC) and the hippocampus^2,3^, the principal areas affected in AD, and not primarily due to neuronal death. While the precise molecular mechanism remains unclear, it is widely accepted that AD-associated synaptopathy is caused by elevated levels of soluble oligomeric β-amyloid (Aβ), which specifically targets synapses and disrupts various signaling molecules and pathways involved in synaptic function^4,5^. A number of candidate molecules have been linked to AD-associated synaptic dysfunction.

Of particular interest are members of the Rho-family of guanosine triphosphatases (GTPases), a subfamily of the Ras superfamily of GTPases^6^, RhoA, Rac1 and Cdc42, which regulate synapses, more specifically dendritic spine morphology and function^7^, by regulating the actin cytoskeleton, the main structural component of dendritic spines. RhoA favors the destabilization of dendritic spines, while Rac1 and Cdc42 promote their stabilization and maturation^8^. Given their critical role in synaptic function, aberrant Rho-GTPase signaling leads to widespread neuronal network dysfunction and has been proposed to play a key role in AD. RhoA subcellular mislocalization and altered levels have been reported in both human AD brains and the human amyloid precursor protein (hAPP) Tg2576 (Swedish mutation) AD mouse model^9,10^. Furthermore, we recently demonstrated that synaptic dysfunction and synapse loss in cultured hippocampal neurons exposed to soluble oligomeric Aβ, and in the J20 hAPP (Swedish and Indiana mutations) AD mouse model^11^, are both preceded and dependent on increased RhoA activity^12^.

Another important molecule implicated in AD-associated synaptic dysfunction is the activity-regulated immediate-early gene Arc/Arg3.1^13^. Arc plays a critical role in synaptic plasticity and memory formation by regulating postsynaptic trafficking of AMPA (α-amino-3-hydroxy-5-methyl-4-isoxazolepropionic acid)-type glutamate receptors at excitatory synapses during long-term potentiation (LTP) consolidation and long-term depression evocation^14,15^. Several lines of evidence have shown Arc levels to be altered in human AD brains, in various AD mouse models, and in cultured hippocampal neurons exposed to oligomeric Aβ^5,16,17^.

How these two critical signaling molecules are dysregulated is currently unknown. However, they share one common regulatory molecule, the ubiquitin-protein ligase E3A, Ube3A/E6-AP^18^. Ube3A is best known for its causative role in the rare neurodevelopmental disorder, Angelman Syndrome (AS). AS is characterized by microcephaly, severe intellectual deficits, abnormal sleep patterns, and hyperactivity, among several others, due to the loss of function of the imprinted UBE3A gene, located on chromosome 15q11.2-q13^19^. Several potential Ube3A substrates have been identified^20^, including ECT2, p53, p27, HR23A, Blk, and interestingly Arc and the RhoA-specific nuclear guanine nucleotide exchange factor (GEF) Ephexin-5, also known as ARHGEF15. One interesting characteristic observed in AS mouse models includes a significant reduction of dendritic spine density and length in neurons of the hippocampus and cortex^21^, akin to AD mouse models. This similarity led to us to investigate whether Ube3A dysfunction was also observed in the Tg2576 AD mouse model.

We found that the levels of Ube3A protein are reduced in the Tg2576 mice in an age-dependent manner, concomitant with a loss of dendritic spine density, and behavioral deficits. Moreover, we show that the decrease in Ube3A protein is neuron specific, and is triggered by elevated Aβ oligomers, which inactivate and decrease the half-life of the Ube3A protein through activation of the non-receptor tyrosine kinase, c-Abl^22^. The reduction of Ube3A protein leads to the accumulation of two of its targets, the synaptotoxic proteins Arc and Ephexin-5, leading to a decrease in surface expression of AMPA receptor subunit GluR1 and an increase in active RhoA, respectively. This culminates in altered synaptic function, and the aberrant pruning of dendritic spines and synapses. Finally, we show that restoring the levels of Ube3A protein in neurons completely protects against the synaptotoxic effects of Aβ oligomers, including the loss of surface-expressed GluR1, and the loss of dendritic spines. Taken together, these data suggest that the loss of Ube3A function plays a critical role in AD pathogenesis, specifically in synaptic dysfunction. Moreover, this study highlights Ube3A as a potential therapeutic target for AD.

## Results

### Ube3A is decreased in Tg2576 hippocampus

**Fig. 1:**
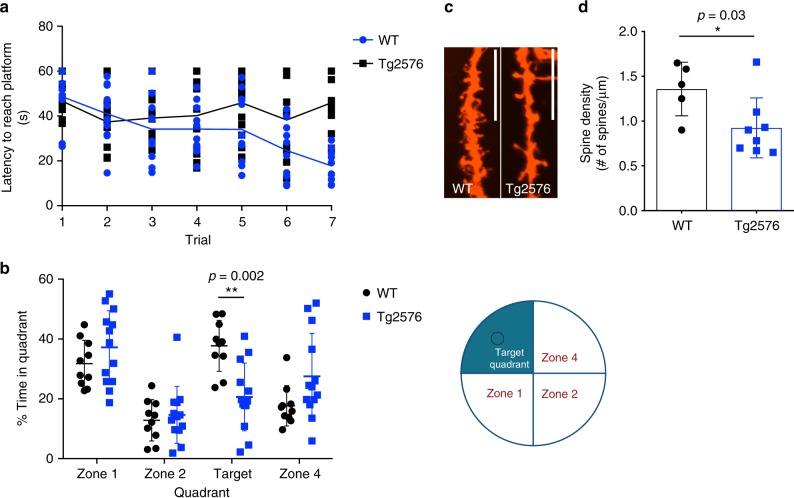
Tg2576 mice show behavioral deficit and dendritic spine abnormalities. **a** Graph of Morris water maze (MWM) 7-day training phase, showing latency to locate a hidden platform (time in seconds) in wild-type (WT) and Tg2576 mice. Data presented as mean values ± standard deviation (s.d.) of *N* = 10 WT and *N* = 13 Tg2576 mice. **b** MWM probe test showing the distribution of time spent (%) in each designated quadrant (illustrated to the right of the graph) in the absence of platform (100% = 60 s). Data presented as mean values ± s.d. of *N* = 10 WT and *N* = 12 Tg2576. **c**, **d** Representative confocal images (**c**) and quantification (**d**) of hippocampal (CA1 region) showing reduced dendritic spine density in Tg2576 relative to WT mice. Scale bar = 12 μM. Data presented as mean values ± s.d. of *N* = 5 WT and *N* = 7 Tg2576. Note that statistical analyses were performed using two-way analysis of variance (ANOVA) followed by Bonferroni post hoc multiple comparisons (**b**) and using unpaired Student’s *t*-test (**d**)

The Tg2576 AD mouse model^9^ overexpresses hAPP carrying the Swedish mutation (KM670/671NL), linked to early-onset familial AD. As previously shown^9,23^, we found that the Tg2576 mice developed amyloid plaques in the hippocampus (Supplementary Fig. 1A, B) and age-dependent progressive behavioral deficits as measured by their inability to learn and to recall the location of a hidden platform in the Morris water maze (MWM) task, evident by 12–14 months of age, compared to wild-type (WT) littermates (Fig. 1a, b). No differences in swimming speeds and ability to locate a visible platform were observed between WT and Tg2576 mice (Supplementary Fig. 1C, D). The behavioral deficits in Tg2576 mice were accompanied by a decrease in dendritic spine density in the CA1 region of the hippocampus, observed by 9–10 months of age (Fig. 1c, d).

**Fig. 2:**
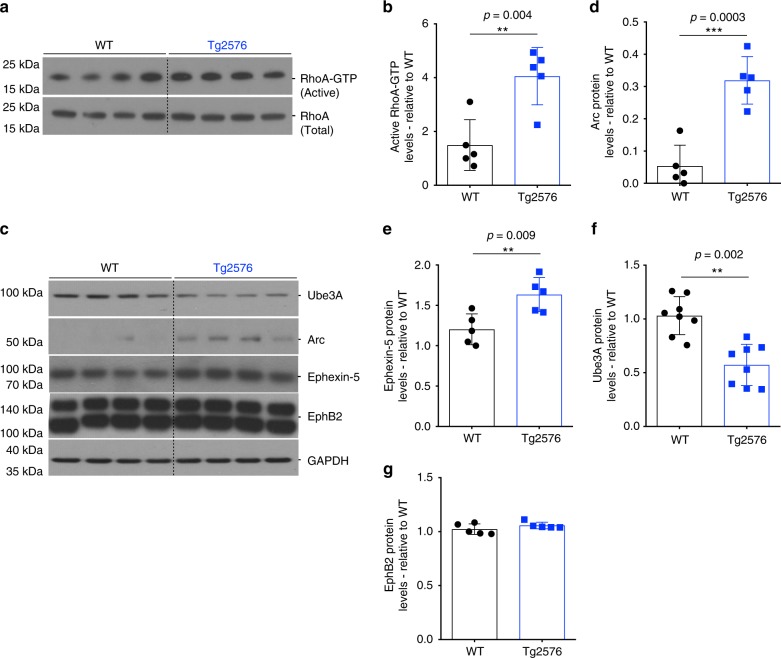
Ube3A is decreased in Tg2576 mice hippocampus. **a**–**c** Representative western blot showing levels of total and active RhoA (**c**), and levels of Ube3A, Arc, Ephexin-5, and EphB2 in wild-type (WT) and Tg2576 mice. **b**–**g** Quantification of western blot data showing elevated levels of active RhoA (**b**), Arc (**d**), Ephexin-5 (**e**), and EphB2 (**g**) in Tg2576 relative to WT mice. **f** Quantification of western blot data showing decreased levels of Ube3A in Tg2576 relative to WT mice. Protein quantifications were normalized to glyceraldehyde 3-phosphate dehydrogenase (GAPDH) as housekeeping gene. Data presented as mean ± s.d. of *N* = 5 WT and *N* = 5 Tg2576 mice. Note that statistical analyses were performed using unpaired Student’s *t*-test for all experiments

We have previously shown that the loss of dendritic spine density in the J20 mouse model^11^ was accompanied by an increase in RhoA activity^12^. To determine whether this was the case in the Tg2576 mice, whole hippocampi were collected at 9–10 months of age to assess RhoA activity. Similar to J20 mice, we found an increase in the levels of active RhoA (RhoA-GTP) in Tg2576 compared to WT mice (Fig. 2a, b). The activity of RhoA, like other GTPases, is regulated by GEFs, which catalyze the exchange of bound guanosine-5′-diphosphate (GDP) for guanosine-5′-triphosphate (GTP), and inactivated by GTPase activating proteins, which catalyze the hydrolysis of GTP to GDP^24^. One of the critical neuronal RhoA-specific GEFs is Ephexin-5, which plays an important role in neuronal development and maturation by negatively regulating synapse formation^25^, and recent evidence suggests that Ephexin-5 may be upregulated in AD brains and in AD mouse models^26^. Similarly, we found an increase in the levels of Ephexin-5 in Tg2576 hippocampi at 9–10 months of age (Fig. 2c–e).

The levels of Ephexin-5 are thought to be regulated through proteasomal degradation following the activation of the erythropoietin-producing hepatocellular (Eph) family of receptor tyrosine kinases, EphB2. This results in ubiquitination and proteasomal targeting of Ephexin-5, catalyzed by Ube3A^25^. Previous studies have shown EphB2 levels to be decreased in the J20 AD mouse model^27^, which might account for elevated levels of Ephexin-5. While we did not observe any differences in the levels of EphB2 protein by western blot in the hippocampi of Tg2576 mice at 9–10 months of age (Fig. 2c–g), we found a decrease in the levels of Ube3A protein (Fig. 2c–f). Depletion of Ube3A protein was evident at 6–7 months of age in Tg2576 mice (Supplementary Fig. 2A–C), and was region specific, observable in the frontal cortex and hippocampus, but not in the cerebellum or olfactory bulb (Supplementary Fig. 2D–F). We also analyzed the levels of Arc, another potential important Ube3A substrates in neurons, which showed an increase in Tg2576 hippocampi compared to WT mice (Fig. 2c, d). Together, these results show an inverse correlation between the levels of Ube3A and those of two of its downstream targets, namely Ephexin-5 and Arc, which we hypothesize might account for the loss of dendritic spine density and synaptic dysfunction observed in the Tg2576 mice.

### Ube3A is decreased in hippocampal neurons in response to Aβ

**Fig. 3:**
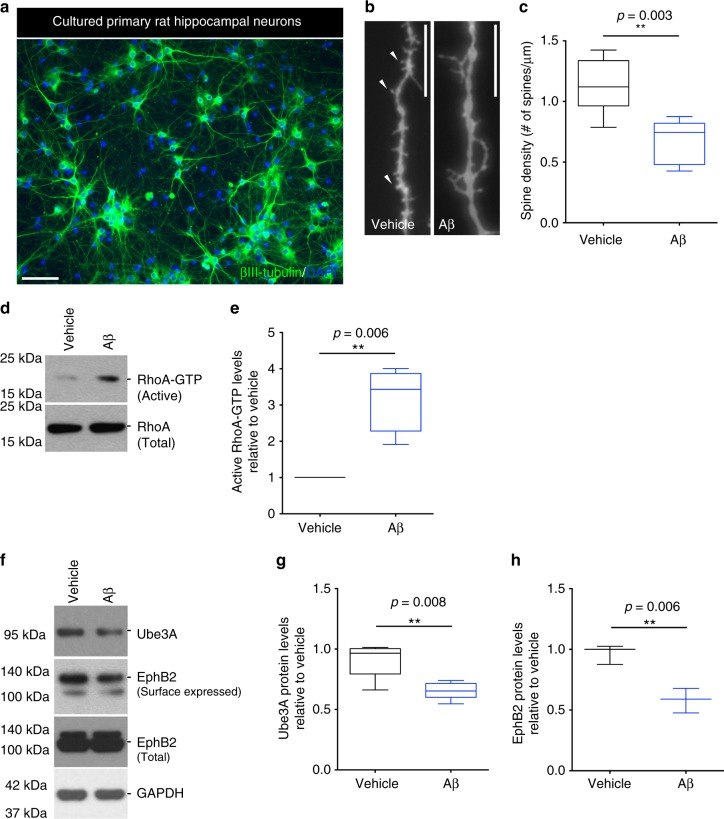
Ube3A is decreased in rat primary hippocampal neurons in the presence of oAβ. **a** Representative image of a hippocampal neuronal culture labeled with βIII-tubulin and counterstained with 4′,6-diamidino-2-phenylindole (DAPI). Scale bar = 500 μM. **b**, **c** Representative confocal images (**b**) and quantification (**c**) of labeled cultured neurons showing decreased dendritic spine density in oAβ-treated (24 h) relative to vehicle-treated cultures. Arrowheads point to examples of mushroom spines. Scale bar = 12 μM. Data presented as mean ± min. to max. values of at least 20 neurons from *N* = 4 independent cultures. **d**, **f** Representative western blot showing levels of total and active RhoA (**d**), and levels of surfaced-expressed and total EphB2, and Ube3A (**f**) in vehicle- and oAβ-treated neurons. **e** Quantification of western blot data showing elevated levels of active RhoA in oAβ-treated (6 h) relative to vehicle-treated cultures. **g**, **h** Quantification of western blot data showing decreased levels of surface-expressed EphB2 in oAβ-treated (24 h) relative to vehicle-treated cultures. Protein quantifications were normalized to glyceraldehyde 3-phosphate dehydrogenase (GAPDH) as housekeeping gene. Data presented as mean ± min. to max. values of at least *N* = 3 independent cultures. Note that statistical analyses were performed using unpaired Student’s *t*-test for all experiments

To further establish a causal relationship between altered levels of Ube3A and those of Arc and Ephexin-5, we decided to use an in vitro model of Aβ_1–42_ synaptotoxicity. Enriched primary rat hippocampal neurons, cultured for 21 days in vitro (DIV; Fig. 3a), were treated with synthetic soluble oligomeric Aβ_1–42_ (referred to as oAβ for the rest of the text; Supplementary Fig. 3A) for 24 h. No cell death was detected under these conditions (Supplementary Fig. 3B); however, consistent with our findings in the Tg2576 mice, a decrease in dendritic spine density was observed (Fig. 3b, c), concomitant with increased levels of active RhoA-GTP (Fig. 3d, e). Moreover, consistent with our findings in the Tg2576 mice, western blot analysis revealed a decrease in the levels of Ube3A protein (Fig. 3f, h). Interestingly, consistent with previous findings^27^, we also detected a decrease in the levels of surface-expressed EphB2 protein (Fig. 3f, g), suggesting that the loss of EphB2 may also contribute to the accumulation of Ephexin-5. Since Ube3A is also expressed in astrocytes^28^, we wanted to determine whether Ube3A levels were also altered in these cells. Contrary to primary hippocampal neurons, treatment of astrocyte-enriched cultures (Supplementary Fig. 4A) with oAβ resulted in a modest increase in Ube3A levels (Supplementary Fig. 4B, C), suggesting that the oAβ-induced decrease in Ube3A is specific to neurons.

### Aβ induces c-Abl-dependent destabilization of Ube3A

**Fig. 4:**
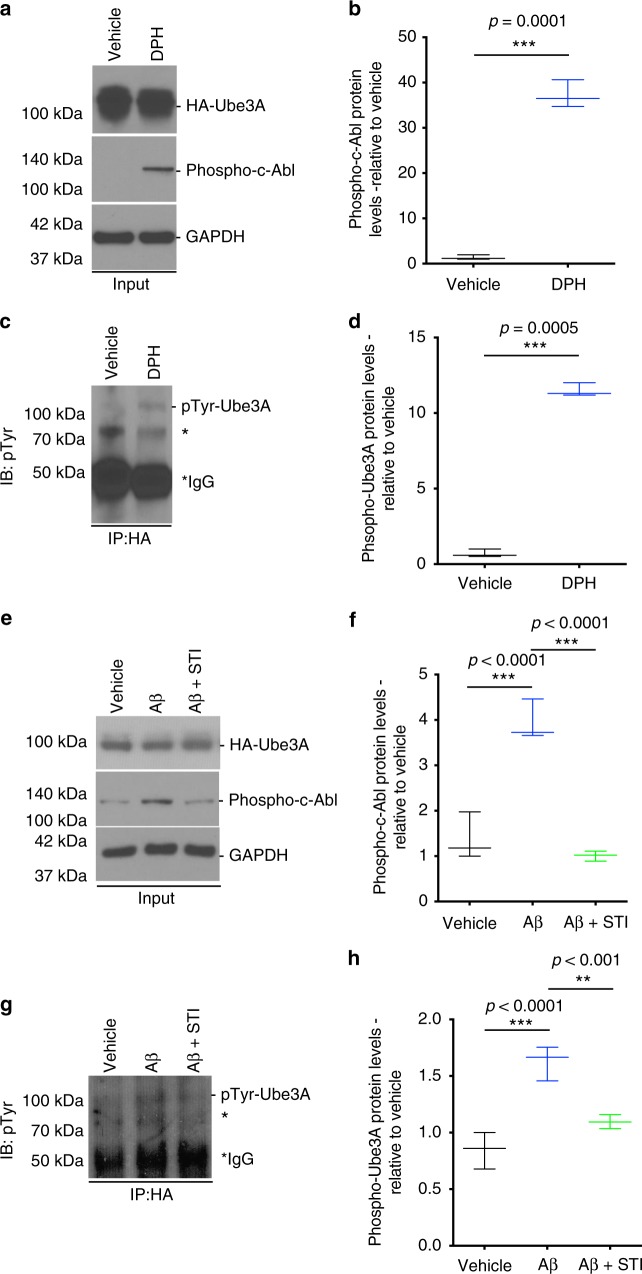
oAβ induces c-Abl phosphorylation and Ube3A destabilization in rat primary hippocampal neurons. **a**–**c** Representative western blot showing levels of HA-Ube3A and phospho-c-Abl in the input fraction, and the levels of phospho-Ube3A (IB:pTyr) in the immunoprecipitated (IP:HA) fraction of transfected B35 cells treated with either vehicle or DPH. **b**–**d** Quantification of western blot data showing elevated levels of phospho-c-Abl (**b**) and phospho-Ube3A (**d**) in DPH-treated (1 h) relative to vehicle-treated cultures. **e**–**g** Representative western blot showing levels of HA-Ube3A and phospho-c-Abl in the input fraction, and the levels of phospho-Ube3A (IB:pTyr) in the immunoprecipitated (IP:HA) fraction of transfected B35 cells treated with either vehicle or oAβ (2 h) in the presence or absence of c-Abl inhibitor STI (1 h pre-treatment). **f**–**h** Quantification of western blot data showing elevated levels of phospho-c-Abl (**b**) and phospho-Ube3A (**d**) in oAβ-treated (6 h) relative to vehicle-treated cultures, and abrogation of this increase by STI (1 h pre-treatment). Protein quantifications were normalized to glyceraldehyde 3-phosphate dehydrogenase (GAPDH) as housekeeping gene. Data presented as mean ± min. to max. values of *N* = 3 independent cultures. Note that statistical analyses were performed using unpaired Student’s *t*-test (**b**, **c**) and one-way analysis of variance (ANOVA) followed by Tukey's post hoc multiple comparisons (**f**–**h**). *Unidentified band

In mammals, the Ube3A gene is subject to genomic imprinting, whereby only the maternal allele is expressed in neurons^29^. The paternal allele is silenced through the expression of a Ube3A-antisense (Ube3A-ATS), transcribed as part of a large non-coding antisense transcript^30^. We first determined whether oAβ affected the levels of Ube3A messenger RNA (mRNA). However, quantitative real-time PCR (qPCR) analysis of oAβ-treated hippocampal neurons for 24 h did not show any differences in the levels of Ube3A mRNA (Supplementary Fig. 5). We hypothesize that oAβ could affect the ligase function of Ube3A. One important regulatory mechanism of Ube3A is through c-Abl-mediated phosphorylation tyrosine residue 636 (Y636), which impairs its E3 ligase activity^31^. c-Abl has previously been implicated in AD pathogenesis, and has been shown to be activated in AD brains and in AD mouse models^32–34^, including in the Tg2576 mice, as we found (Supplementary Fig. 6A, B). Since phospho-Ube3A antibodies are not available, we decided to test our hypothesis in B35 neuroblastoma cells^35^ overexpressing hemagglutinin (HA)-tagged Ube3A for immunoprecipitation. Consistent with our hypothesis, we found that treatment of these cells with either the c-Abl activator, DPH^36^, used as a positive control (Fig. 4a–d), or with oAβ (Fig. 4e–h) resulted in an increase in phospho-tyrosinated Ube3A (pTyr-Ube3A), as early as 1 h, as determined by immunoblotting analysis of immunoprecipitated HA-Ube3A with a phospho-tyrosine-specific antibody. To confirm the involvement of c-Abl in tyrosine-phosphorylation of Ube3A, cells were pre-treated with the specific c-Abl inhibitor STI-571^37^ for 1 h prior to oAβ treatment. Inhibition of c-Abl resulted in the complete abrogation of Ube3A phosphorylation by oAβ (Fig. 4g, h).

**Fig. 5:**
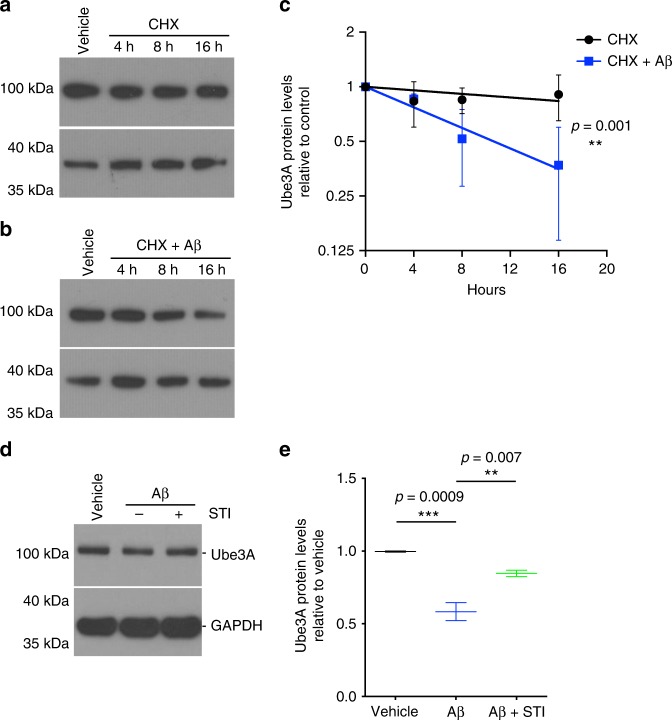
oAβ increases the depletion of Ube3A. **a**, **b** Representative western blots showing the degradation of Ube3A protein over time (4, 8, and 16 h) in cycloheximide (CHX)-treated (1 h pre-treatment) neuronal cultures exposed to vehicle or oAβ. **c** Quantification of western blot data showing increased degradation rate of Ube3A in neuronal cultures after oAβ treatment relative to vehicle; half-life of ~61 h for vehicle vs. ~10.5 h for oAβ. Data presented as mean ± s.d. of 3 independent cultures. Note that statistical analyses were performed using non-linear fit of exponential decay (rejected null hypothesis = *K* same for all data sets; preferred model = *K* different for each data set). **d** Representative western blot showing levels of Ube3A of neuronal cultures treated with either vehicle or oAβ in the presence or absence of c-Abl inhibitor STI. **e** Quantification of western blot data showing decreased Ube3A protein in oAβ-treated (24 h) relative to vehicle-treated cultures, abrogated by treatment with c-Abl inhibitor STI (1 h pre-treatment). Protein quantifications were normalized to glyceraldehyde 3-phosphate dehydrogenase (GAPDH) as housekeeping gene. Data presented as mean ± min. to max. values of *N* = 3 independent cultures. Note that statistical analyses were performed using one-way analysis of variance (ANOVA) followed by Tukey's post hoc multiple comparisons

Interestingly, our results suggest that tyrosine-phosphorylation of Ube3A results in destabilization and decrease in the half-life of the protein. Under baseline conditions, Ube3A protein has a half-life of ~61 h, as determined by cycloheximide (CHX) chase assay (Fig. 5a–c). However, treatment with oAβ increases the degradation rate of the Ube3A protein, decreasing its half-life to ~10.5 h (Fig. 5b, c). The decrease in Ube3A protein was completely blocked by pre-treatment with STI-571, consistent with the role of c-Abl in mediating Ube3A inactivation and degradation (Fig. 5d, e). These results are consistent with previous studies which showed that STI-571 treatment completely blocked the synaptotoxic effects and the loss of dendritic spines induced by oAβ^34^.

### Depletion of Ube3A correlates with increased Arc and Ephexin-5

**Fig. 6:**
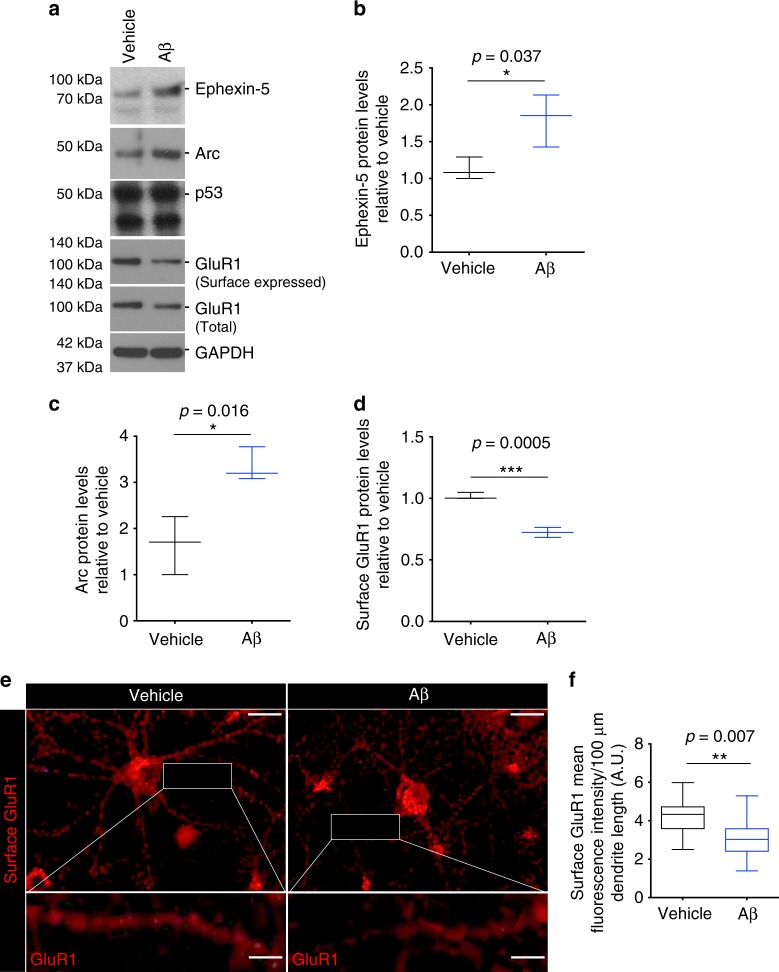
oAβ dysregulates Ube3A downstream proteins in rat hippocampal neurons. **a** Representative western blot showing levels of Ephexin-5, Arc, surfaced-expressed and total GluR1, and p53 in vehicle- and oAβ-treated neuronal cultures. **b**–**d** Quantification of western blot data showing increased levels of Ephexin-5 (**b**) and Arc (**c**), and decreased levels of surface-expressed GluR1 (**d**) in oAβ- (8–10 h) relative to vehicle-treated neurons. Data presented as mean ± min. to max. values of *N* = 3 independent cultures. **e** Representative confocal images of neurons treated with and without oAβ (8–10 h), labeled with GluR1. Scale bar = 50 μM. Rectangle shows a higher magnification detail of GluR1-positive spines. Scale bar = 10 μM. **f** Quantification of surface GluR1 fluorescence intensity showing decreased surface GluR1 in oAβ- relative to vehicle-treated neurons. Data presented as mean ± min. to max. values/100 μM dendrite length of *N* = 3 independent cultures. Note that statistical analyses were performed using unpaired Student’s *t*-test for all experiments

As we observed in the Tg2576 mice hippocampi, the levels of Ube3A inversely correlated with the levels of Arc and Ephexin-5. To determine whether this correlation was also observed in hippocampal neurons, they were treated with Aβ for 8–10 h, and whole-cell lysates were collected for western blot analysis. This revealed an increase in the levels of both Ephexin-5 and Arc protein (Fig. 6a–d). Intriguingly, analysis of another candidate Ube3A substrate, p53, did not show any altered levels after Aβ treatment (Fig. 6a).

The consequences of upregulation of Arc and Ephexin-5 are well characterized. In the case of Arc, one of its key function is to regulate postsynaptic trafficking of AMPA receptor subunits, and to regulate spine morphology and network stability^14,15,38^. Aberrant upregulation of Arc has been shown to induce a decrease in surface expression of the AMPA receptor subunit GluR1^39^. As previous studies have shown, when hippocampal neurons were treated with Aβ, inactivation of Ube3A correlated with a decrease in the levels of surface-expressed GluR1 subunit, as determined by western blot analysis of isolated biotinylated surface GluR1 (Fig. 6a–d), and by immunostaining of surface GluR1 (Fig. 6e, f).

**Fig. 7:**
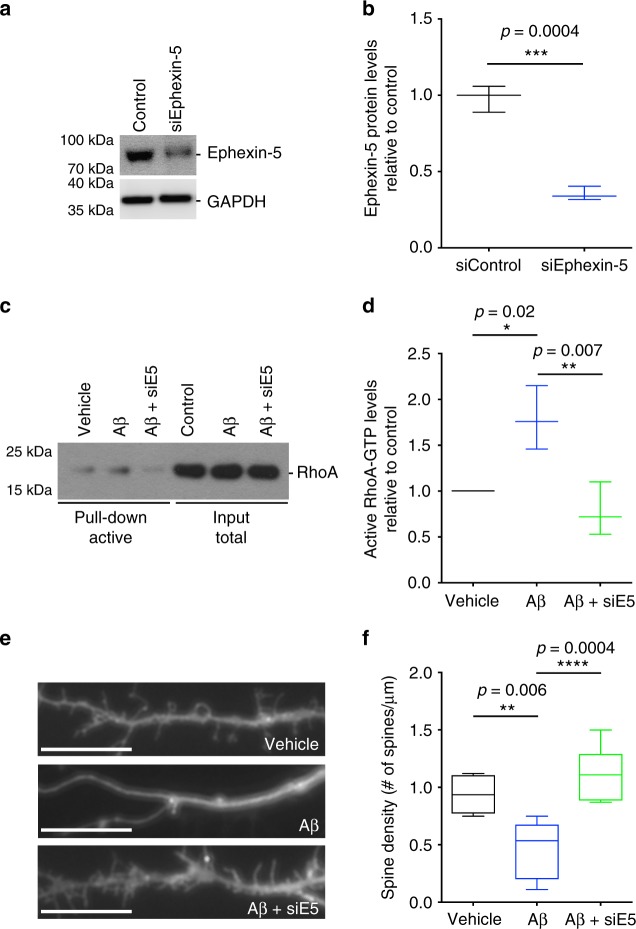
oAβ-induced RhoA activation is Ephexin-5 dependent. **a** Representative western blot showing levels of Ephexin-5 in control and siEphexin-5-treated neuronal cultures. **b** Quantification of western blot data showing depletion of Ephexin-5 protein in siEphexin-5-treated (24 h) relative to siScrambled-treated control neurons. Data presented as mean ± min. to max. values of *N* = 3 independent cultures. Note that statistical analyses were performed using unpaired Student’s *t*-test. **c** Representative western blot showing levels of total and active in vehicle- and oAβ-treated neurons in the presence or absence of siEphexin-5. **d** Quantification of western blot data showing elevated levels of active RhoA in oAβ-treated (6 h) relative to vehicle-treated cultures, and abrogation of RhoA activation after siEphexin-5 treatment. Data presented as mean ± min. to max. values of *N* = 3 independent cultures. Note that statistical analyses were performed using one-way analysis of variance (ANOVA) followed by Tukey's post hoc multiple comparisons. **e**, **f** Representative confocal images (**e**) and quantification (**f**) of labeled cultured neurons showing decreased dendritic spine density in oAβ-treated (24 h) relative to vehicle-treated cultures and abrogation of this decrease by siEphexin-5. Scale bar = 12 μM. Data presented as mean ± min. to max. values of at least 20 neurons from *N* = 3 independent cultures. Note that statistical analyses were performed using one-way ANOVA followed by Tukey's post hoc multiple comparisons

Ephexin-5, on the other hand, is a key RhoA-specific GEF in neurons, whose activity is critical for spine formation during development^25^. Upregulation of Ephexin-5 leads to aberrant RhoA activation and loss of dendritic spine density. We have shown that oAβ leads to increased RhoA activation in neurons (Fig. 3d, e). To confirm that Ephexin-5 is responsible for upregulation of RhoA activity in our model, we depleted the levels of Ephexin-5 by Penetratin-1-linked small interfering RNA (siRNA)^40^ in cultured hippocampal neurons for 24 h (Fig. 7a, b), prior to treating them with oAβ for 8 h. Depletion of Ephexin-5 completely blocked the oAβ-induced upregulation of RhoA activity (Fig. 7c, d). Moreover, downregulation of Ephexin-5 completely rescued the loss of dendritic spine normally observed in hippocampal neurons treated with oAβ after 24 h (Fig. 7e). These data suggest that increased RhoA activity is dependent on the upregulation of Ephexin-5. We also assessed the levels of two other RhoA-specific GEFs, namely Ephexin-1^41^ and Lfc^42^. We did not observe any differences in the levels or activity of Ephexin-1 (Supplementary Fig. 7A, B) while, intriguingly, we found a decrease in the activity of Lfc (Supplementary Fig. 7C, D). This is consistent with the idea that the activation of RhoA is mediated by Ephexin-5 in neurons after exposure to oAβ.

### Restoring Ube3A levels protects against oAβ-induced synaptotoxicity

To confirm that the loss of Ube3A function plays a key role in mediating oAβ-induced synaptic dysfunction in neurons, we decided to restore the levels of Ube3A protein through lentiviral-mediated upregulation of a Ube3A. To carefully regulate transgene expression in neurons, the Ube3A construct was fused with a destabilizing domain (DD), derived from the *Escherichia coli* dihydrofolate reductase (Supplementary Fig. 8A), which results in fast proteasomal degradation of the entire fusion protein in the absence of a stabilizing agent^43^, in this case the small molecule trimethoprim (TMP). Once TMP is added, it binds to DD and stabilizes the fused protein, allowing it to accumulate in the cell. In our system, infection of hippocampal neurons with a DD-Ube3A-expressing lentivirus resulted in undetectable expression of exogenous Ube3A in the absence of TMP. However, the addition of TMP to the culture medium resulted in a dose-dependent increase in DD-Ube3A after 24 h (Supplementary Fig. 8B, C).

**Fig. 8:**
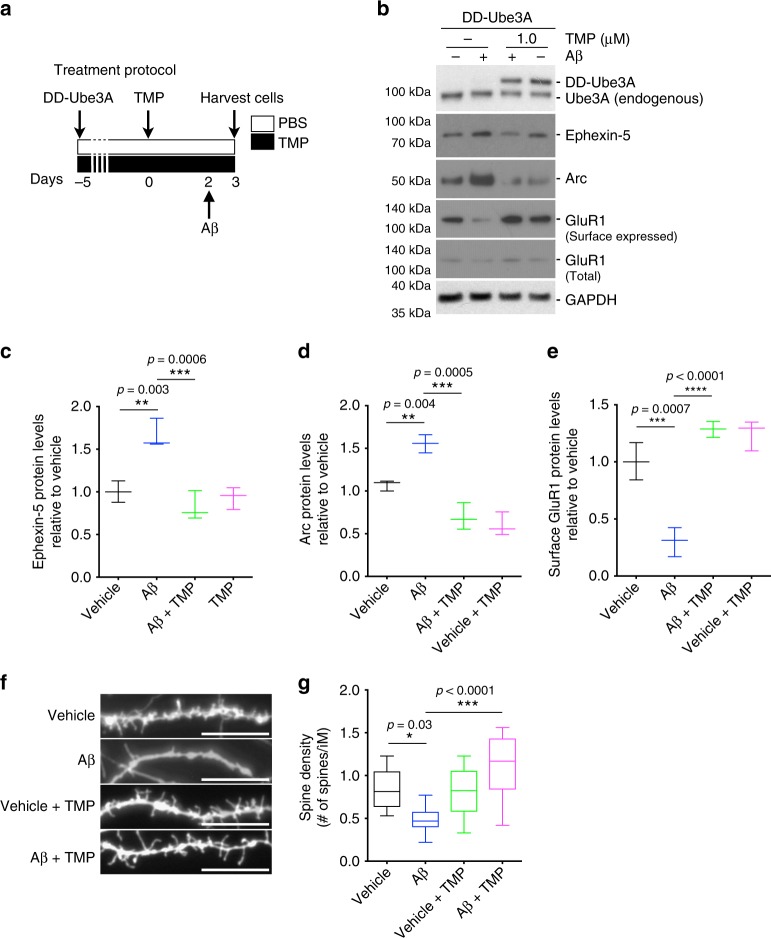
Restoration of Ube3A proteins levels protects neurons against the synaptotoxic effects of oAβ. **a** Timeline of the treatment protocol: 5 days after DD-Ube3A lentiviral infection, neurons were treated with TMP (trimethoprim) or vehicle (day 0), after 2 days, oAβ (or Vehicle) was added to the media, and on day 3 neurons were harvested. **b** Representative western blot showing levels of DD-Ube3A, Ephexin-5, Arc, and surface-expressed and total GluR1 in neuronal cultures treated with oAβ or vehicle in the presence or absence of Ube3A overexpression. **c**, **d** Quantification of western blot data showing increased levels of Ephexin-5 (**c**) and Arc (**d**) in oAβ-treated (24 h) relative to vehicle-treated cultures, and abrogation of this increase by Ube3A overexpression. **e** Quantification of western blot data showing decreased levels of surface GluR1 (**f**) in oAβ-treated (24 h) relative to vehicle-treated cultures, and abrogation of this decrease by Ube3A overexpression. Data presented as mean ± min. to max. values of *N* = 3 independent cultures. **f**, **g** Representative confocal images (**f**) and quantification (**g**) of labeled cultured neurons showing decreased dendritic spine density in oAβ-treated (24 h) relative to vehicle-treated cultures and abrogation of this decrease by Ube3A overexpression. Scale bar = 12 μM. Data presented as mean ± min. to max. values of at least 20 neurons from *N* = 3 independent cultures. Note that statistical analyses were performed using one-way analysis of variance (ANOVA) followed by Tukey's post hoc multiple comparisons

To test whether upregulation of Ube3A could rescue the effects of oAβ, cultured hippocampal neurons at 14 DIV were infected with DD-Ube3A-expressing lentivirus for 5 days, followed by TMP treatment for 48 h. The neurons were then treated with oAβ for 24 h, and whole-cell lysates prepared for western blot analysis or fixed for microscopy (Fig. 8a). Upregulation of Ube3A in oAβ-treated neurons completely blocked the upregulation of Arc and Ephexin-5 (Fig. 8b–d). Preventing upregulation of Arc also completely blocked the loss of surface GluR1 by oAβ (Fig. 8b–e). Additionally, dendritic spine analysis in fixed neurons revealed that prevention of Ephexin-5 induction resulted in a complete protection against oAβ-induced loss of dendritic spine density (Fig. 8f, g). These data strongly indicate that depletion of Ube3A below a critical threshold is required for the accumulation of the synaptotoxic proteins Arc and Ephexin-5, and synaptic dysfunction by oAβ.

## Discussion

While widespread synaptic dysfunction and the loss of synapses are invariable features of AD, the exact mechanism(s) driving these changes remains unclear. Accumulating evidence suggests that aberrant signaling by two key synaptic proteins may play an important role in AD-associated synaptopathy, namely Arc/Arg3.1 and Ephexin-5, whose levels are anomalously upregulated in the brain of AD patients, and in various AD mouse models^5,16,17,25,26^. The exact mechanism(s) leading to the upregulation of these two proteins is not fully understood. However, several prior studies suggest that oligomeric Aβ may be at least partially responsible, as it can induce their rapid expression in neurons^5,16,17,44^.

Herein we show that degradation of both Arc and Ephexin-5 may also be impaired in AD, perhaps contributing to their sustained expression in neurons exposed to oligomeric Aβ. In this report we show that the AS-associated protein, Ube3A, a key E3 ligase regulating the degradation of Arc and Ephexin-5^21,25,45^, is downregulated in cognitively impaired AD transgenic mouse brains, and in cultured primary rat hippocampal neurons treated with oligomeric Aβ, suggesting that Ube3A may also be a key player in AD-associated synaptic dysfunction. Consistent with this idea, the observed depletion of Ube3A paralleled the oAβ-induced increase in both Arc and Ephexin-5. Their accumulation can lead to decreased surface-expressed GluR1 and to the loss of dendritic spine density, respectively^25,26,39^. More importantly, restoration of Ube3A in oAβ-treated neurons was sufficient to completely block the induction of Arc and Ephexin-5, while also abrogating the loss of surface-expressed GluR1 and dendritic spine density, confirming the contribution of Ube3A to these processes. Interestingly, our data indicate that the decrease in Ube3A in response to oAβ exposure appears to be specific to neurons, as Ube3A levels were conversely increased in cultured primary rat astrocytes, suggesting a differential response to oAβ between neurons and astrocytes. Indeed, prior studies have shown that astrocytes, unlike neurons, are resistant to oAβ cytotoxicity, possibly due to the activation of distinct signaling pathways^46,47^.

**Fig. 9:**
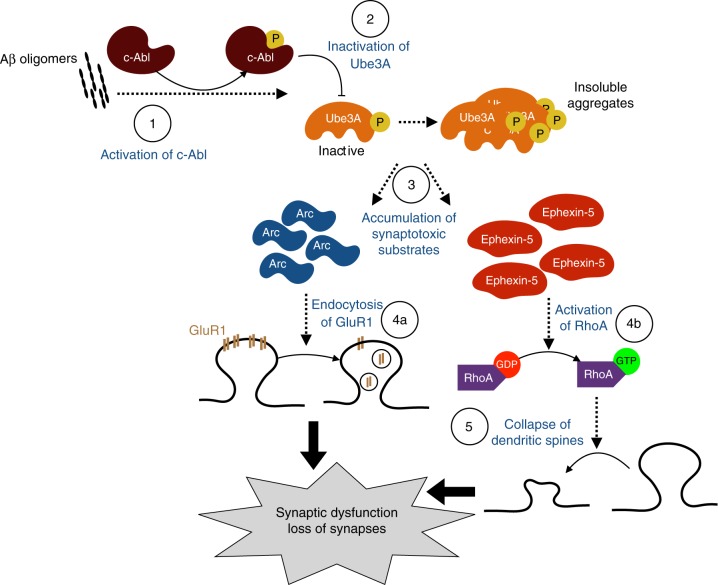
Diagram of the model of Ube3A in Alzheimer’s disease (AD) pathology. Exposure of oAβ in neurons induces the phosphorylation and activation of c-Abl (1), which in turn phosphorylates and inactivates Ube3A (2), causing the protein to become insoluble and aggregate. (3) Depletion of Ube3A allows for the accumulation of two of its substrates, Arc and Ephexin-5. (4a) The accumulation of Arc leads to the aberrant internalization of AMPA receptor subunit GluR1, impairing synaptic function, while (4b) Ephexin-5 accumulation leads to the persistent activation of RhoA, resulting in synaptic pruning

One important signaling protein that we found to be involved in the downregulation of Ube3A is c-Abl, whose activity was upregulated in impaired Tg2576 mice. c-Abl has previously been implicated in various aspects of AD pathogenesis, ranging from aberrant APP processing and tau phosphorylation, loss of dendritic spines, and neuronal apoptosis^32–34,48,49^. Our results show that activation of c-Abl by oAβ, or the c-Abl activator DPH, triggers the phosphorylation and inactivation of Ube3A, resulting in an unstable form of the protein, thereby reducing its half-life from over 24 h to about 8 h, with no effects on its transcription. Treating the neurons with the c-Abl-specific inhibitor, STI-571, completely blocked the phosphorylation and degradation of Ube3A. Interestingly, consistent with these results, this inhibitor has previously been shown to completely block the oAβ-induced loss of dendritic spine density and impaired LTP in vitro in cultured neurons^34^, and prevent tau phosphorylation in vivo and behavioral impairments in an AD mouse model^50^. Taken together, we propose a model (Fig. 9) whereby oligomeric Aβ leads to the phosphorylation and activation of c-Abl, which in turn phosphorylates and inactivates Ube3A, resulting in an unstable protein which is then degraded. Depletion of Ube3A thus allows the accumulation of the downstream targets, Arc and Ephexin-5, which results in the aberrant endocytosis of GluR1 and activation of RhoA, culminating in widespread synaptic dysfunction and loss of dendritic spines.

An intriguing remaining question from these studies relates to the mechanism regulating the degradation of Ube3A after its inactivation by c-Abl. Ube3A has been suggested to target itself for degradation^20,51^, and it is well established that Ube3A can associate with the proteasome to regulate its function^52–54^. How then can a loss-of-function post-translational modification by c-Abl lead to its degradation? One possible explanation is that, at least in this context, the degradation of Ube3A is not mediated by the proteasome. Indeed, neither proteasomal nor lysosomal inhibitors were able to prevent the depletion of Ube3A protein by oAβ (Supplementary Fig. 5B). Intriguingly however, our results suggest that phosphorylation and inactivation of Ube3A by c-Abl may result in its aggregation and deposition. In support of this hypothesis, we were able to detect elevated levels of Ube3A in insoluble fractions prepared from Tg2576 mice (Supplementary Fig. 6D), consistent with previous findings^55^. Further supporting this hypothesis is the fact that Ube3A function has been shown to be highly susceptible to oxidative stress, which renders it insoluble^56^, and the fact that c-Abl is activated as a result of oxidative stress^57,58^. Together, these suggest that Ube3A is made insoluble after its phosphorylation and inactivation by c-Abl downstream of oAβ. However, more research is needed to fully elucidate this process.

The consequences of loss of function of Ube3A have been well documented in the context of Angelman syndrome^19^, and there is growing evidence that gain of function of Ube3A may also play a role in autism spectrum disorder (ASD)^52,59–61^. As such, there is tremendous interest in identifying the direct and indirect substrates of Ube3A^20^. This is particularly important for AD research, not only in light of the results of the studies herein, but due to the fact the Ube3A may also influence other aspects of AD pathogenesis, particularly APP and Aβ production. There is solid evidence for this idea. First, AS patients have significantly elevated plasma Aβ40 and 42 compared to age-matched control individuals^62^. Second, aberrant APP regulation has been reported in models of chromosome 15q11–13 duplication, in which Ube3A gene expression is increased 1.5- to 2-fold, which demonstrated that APP levels were decreased 10-fold relative to control samples^63^. Third, post-mortem analysis in 15q11–13 duplication (associated with ASD) shows that neurons from control patients showed higher deposition rates than neurons from subjects with ASD^64^. Fourth, Arc itself has been suggested as a key regulator of APP and BACE1 trafficking and Aβ production^16^. Taken together, these data suggest that Ube3A dysfunction may be intricately linked to AD pathogenesis.

However, compiling a comprehensive list has not been without its share of difficulties and conflicting results. A recent study has demonstrated that disruption of Ube3A function may have widespread inhibitory effects on proteolytic activity of the proteasome^54,65^, potentially resulting in numerous indirect substrates. In fact, while the expression of Arc does change in response to Ube3A, it may be an indirect substrate of Ube3A, highlighted by fact that varying results have been obtained by different groups regarding Arc as a direct Ube3A substrate^21,45,66,67^. Even previously well-established substrates, such as p53, have come into question, due to the fact that Ube3A does not ubiquitinate p53, at least in vitro^45,54^, and to the fact that in our studies p53 levels remained unaltered after Ube3A depletion. Nevertheless, the phenotypic similarities between AS and other neurodevelopmental disorders, and now with AD, can probably be explained by the significant overlap in downstream signaling pathways, which suggest that therapeutic strategies developed for one disorder may also be applicable to others, especially to correct like-phenotypes. More importantly, our results squarely place Ube3A as a prime target for future AD therapeutics.

## Methods

### Materials

All chemicals used were of the highest grade available. Antibodies used in this study include the following (Supplementary Table 1, Supplementary References 1): rabbit anti-Ube3A (Cell Signaling, 1:1000), mouse anti-RhoA (Santa Cruz, 1:1000; Cell Signaling, 1:1000), mouse anti-Arc (Santa Cruz, 1:1000), mouse anti-GluR1 (Millipore, 1:1000 for immunofluorescence), rabbit rabbit anti-GluR1 (Cell Signaling, 1:1000 for western blot), anti-Ephexin-5 (Pierce and Abcam, 1:1000), rabbit anti-Lfc (Cell Signaling, 1:1000), rabbit anti-Ephexin-1 (ECM, 1:500), rabbit anti-phospho-c-Abl (Cell Signaling, 1:1000), rabbit anti-GAPDH (Cell Signaling, 1:2000), rabbit anti-HA (Cell Signaling, 1:1000 for western blot, 1:100 for immunprecipitation), rabbit anti-PSD95 (Cell Signaling, 1:1000), rabbit anti-EphB2 (Millipore, 1:1000), Mouse anti-P53 (Santa Cruz, 1:1000), and mouse anti-APP (Millipore, 1:2000). Reagents used in the study include: Neurobasal medium (ThermoFisher Scientific), B27 supplement (ThermoFisher Scientific), Glutamax (ThermoFisher Scientific), poly-d-lysine (PDL) hydrobromide mol wt > 300,000 (Sigma Aldrich), Dubelcco’s modified Eagle’s medium (DMEM) high glucose (ThermoFisher Scientific), DMEM/F12 (ThermoFisher Scientific), fetal bovine serum (FBS, ThermoFisher Scientific), Tris(2-carboxyethyl)phosphine hydrochloride (TCEP, Sigma Aldrich), Complete Mini protease inhibitor cocktail (Roche Applied Science), Halt protease and phosphatase inhibitor (ThermoFisher Scientific), TRIzol reagent (Ambion), anhydrous dimethyl sulfoxide (DMSO, Sigma Aldrich), methylene chloride (Sigma Aldrich), hexafluoro-2-propanol (HFIP; Sigma Aldrich), 1,1′-Dioctadecyl-3,3,3′,3′-Tetramethylindocarbocyanine Perchlorate (DiI, ThermoFisher Scientific), phosphate-buffered saline pH 7.4 (PBS, ThermoFisher Scientific), tris-buffered saline pH 7.4 (TBS, ThermoFisher Scientific), Superblock (TBS) blocking buffer (ThermoFisher Scientific), paraformaldehyde 16% (PFA, ThermoFisher Scientific), Triton X-100 (ThermoFisher Scientific), radioimmunoprecipitation assay (RIPA) buffer (ThermoFisher Scientific), mammalian protein extraction reagent (M-PER, ThermoFisher Scientific), and DNase/RNase-free water (ThermoFisher Scientific). Extraction buffer (EB) recipe: 20 mM [(4-[2-hydroxyethyl]−1-piperazineethanesulfonic acid]) (HEPES)], pH 7.4, 100 mM NaCl, 20 mM NaF, 1 % Triton X-100, 1 mM sodium orthovanadate, 5 mM EDTA.

### Primary hippocampal neuronal cultures

Hippocampal neuron cultures from both male and female rat embryos (E17–18) were prepared following a slightly modified version of the method of Brewer et al.^68^. Hippocampal neurons were kept in culture at 37 °C with 5% CO_2_ in Neurobasal medium with B27 supplement and Glutamax, and plated at a density of 2.5 × 10^5^ cell/ml on dishes coated with PDL. For our experiments, neurons were used after 14–21 DIV. Rat neuroblastoma B35 cells were cultured and maintained in DMEM high glucose supplemented with 10% FBS and Glutamax.

### Penetratin-1-mediated delivery of siRNA

Oligonucleotide sequences were custom-ordered (Dharmacon) with a protected thiol functionality at the 5′-end (Ephexin-5 siRNA sequences: Sense_S-S.GUCCUUUCUCCGUGUAUGUUU; Antisense_ACAUACACGGAGAAAGGACUU). Oligonucleotide stocks were solubilized in DNase/RNase-free water at a 10 mM concentration. The delivery peptide Penetratin-1 (MP Biomedicals) was cross-linked via a Cys–Cys bond to the desired oligonucleotide as previously described^40^. Briefly, 20 nmole of siRNA was dissolved in 25 μl DNase/RNase-free water with 1.1 μl TCEP (17.6 mM) and incubated at room temperature for 1 h. Then, 25 μl of Penetratin-1 (dissolved in DNase/RNase-free water to 2 mg/ml) was added to the mixture with an additional 180 μl DNase/RNase-free water, and incubated for 15 min at 65 °C for 15 min, and then for 1 h at 37 °C. To visualize nucleic acid, 2 μl of the mixture was separated on 20% TBE gel (ThermoFisher Scientific) and stained with SYBR gold (ThermoFisher Scientific, 1:2500) for 15 min at room temperature.

### Transgenic mice

The Tg2576 mouse model^9^ used in this study was originally generated by Karen Hsiao and overexpresses a mutant form of *APP* (isoform 695) with the Swedish mutation (KM670/671NL). Male mice were kept under standard housing conditions (12 h light/dark cycle) with access to food and water. Age-matched WT littermates were used as controls. All experiments involving animals were performed under the guidelines of the Columbia University Institutional Animal Care and Use Committee.

### Morris water maze

Mice were trained to find a hidden platform in a circular pool of 1.10 m diameter located in a room with extra maze cues. The location of the platform (14 mm diameter) was constant for each mouse during training and was placed 1 cm beneath the surface of the water, maintained at 24 °C throughout the duration of the test. At the end of test, the mice were dried off and placed in a clean cage with extra paper towels to prevent hypothermia. Animals were then monitored inside their cage until eating, drinking, and ambulating normally.

Mice were trained in sessions consisting of four trials a day and for seven consecutive days. Mice started from different quadrants on a random basis during each trial and all four quadrants were used on any given day. The maximum swimming time allowed on each trial was 60 s; if the mouse failed to reach the platform within 60 s, it was manually guided to the platform and kept there for 15 s.

Probe trials (1 session: 1 trials/session) were conducted 24 h after the last training trial. During the probe test the platform was removed and mice were free to swim in the pool for 60 s. Time spent in each quadrant was measured. Trial and probe test sessions were recorded and analyzed with a video tracking system (Noldus). After the probe test, mice were tested for their ability to locate a visible platform in order to exclude differences in vision, swim speed, and motivation. These were performed for two consecutive days, 2 sessions/day with 3 trials/session (its location varying between the three trials of the same session). The times to locate the visible platform and the swim speeds were collected.

### Oligomeric oAβ preparation

Lyophilized oAβ (rPeptide) was dissolved to 1 mM in HFIP for 1 h in a tissue culture hood. The dissolved peptide solution was then aliquoted into Teflon-coated tubes and HFIP was allowed to evaporate at room temperature overnight under sterile conditions. The resulting pellet was further dried in a SpeedVac for 1 h at room temperature. Tubes were sealed and stored at −30 °C freezer with desiccator pellets. To prepare oligomeric oAβ, the pellet was resuspended in DMSO at 5 mM and sonicated for 15 min at room temperature. The solution was further diluted in DMEM/F12 to 100 μM and allowed to oligomerize at 4 °C for 24 h.

### Immunofluorescence and diolistic labeling of neurons

Cells were fixed in 4% PFA in PBS for 10 min at room temperature. Permeabilization was performed using 0.4% Triton X-100 in PBS for 20 min. Then, cells were incubated with the appropriate primary antibody in Superblock with 0.1% Triton X-100 for 12 h at 4 °C, followed by incubation in goat anti-mouse Alexa 568 in Superblock with 0.1% Triton X-100 for 1 h at room temperature. The protocol for diolistic labeling of neurons was adapted from Gan et al.^69^. Briefly, a slurry of tungsten particles (1.1 µm diameter, 100 mg; Bio-Rad) was prepared with DiI stain (5.0 mg) dissolved in methylene chloride, spread on a glass cover slide, and allowed to dry for 15 min. Dye-coated particles were resuspended in water (10 ml) and passed through a Tefzel tube (Bio-Rad) coated with 10 mg/ml solution of polyvinylpyrrlidone and allowed to dry for 1 h at room temperature. Particles were delivered into non-permeabilized PFA-fixed cells using the Helios gene gun system (Bio-Rad), the dye was allowed to diffuse overnight, and neurons were mounted onto glass slides using Gel Mount (Biomeda) for visualization.

### Spine imaging, quantification, and statistics

Labeled neurons were imaged using the Laser Scanning Microscope 510 Meta confocal microscope (Zeiss), equipped with 40× 1.3 NA and 100× 1.4 NA oil-immersion objectives. The NIH Image software program ImageJ was used the quantify DiI-labeled neurons. An average of 12 compressed images (20 µm thick) consisting of pyramidal neurons were quantified for each treatment. Spines were counted with NeuronStudio software in a blinded manner and spine density was represented in number of spines per μm of dendrite length.

### Immunoprecipitation (IP)

Immunoprecipitations were performed using the Dynabeads Protein G IP kit (ThermoFisher Scientific). Briefly, cells were homogenized and incubated overnight at 4 °C with 1.0 μg of HA antibody or mouse IgG as a negative control. Immunocomplexes were captured by incubating with the magnetic beads on a rotator for 2 h at 4 °C and resuspended in cell lysis buffer with LDS-sample buffer for western blotting analysis.

### Sample preparation for immunoblotting analysis

Whole frozen mouse hippocampi, human brain specimens, and cultured cells were homogenized in RIPA buffer supplemented with protease and phosphatase inhibitor cocktail using a Teflon dounce homogenizer and centrifuged at 14,000 rpm for 10 min at 4 °C. Supernatants were collected, normalized using a BCA assay kit (ThermoFisher Scientific), and prepared for loading using 4× sample buffer (ThermoFisher Scientific) and dithiothreitol 10× (ThermoFisher Scientific).

### Insoluble fraction preparation

Insoluble Ube3A protein extracts were prepared from frozen mouse brains as previously described^70^. Briefly, frozen brains were homogenized in a dounce homogenizer in 10 volumes (wt/vol) of EB buffer supplemented with protease inhibitor cocktail and 2 mM phenylmethane sulfonyl fluoride. Resulting homogenates were centrifuged (3000 × *g*, 4 °C, 5 min) and the supernatants collected for total protein analysis. For sarkosyl-soluble and -insoluble fractions, homogenates were centrifuged (27,000 × *g*, 4 °C, 20 min) and the pellet was resuspended in sucrose buffer (0.8 M NaCl and 10% sucrose; 10 ml/g of initial tissue weight) and centrifuged again (27,000 × *g*, 4 °C, 20 min). The resulting supernatants were incubated on a rotator in 1% sarkosyl buffer for 1 h at 25 °C and centrifuged (100,000 × *g* for 2 h) to collect the pellet containing the insoluble proteins.

### Quantitative real-time PCR

RNA from neurons was extracted using TRIzol following the manufacturer’s recommended protocol. Concentration and purity were assessed by measuring the O.D. at 260 and 280 nm with a NanoDrop (ThermoFisher Scientific) and 1 μg was used to synthesize complementary DNA (cDNA) using a first-strand cDNA synthesis kit (Origene) following the manufacturer’s protocol. qPCR was performed using the FastStart SYBR Green Master Mix (Roche Applied Science) on an Eppendorf Realplex Mastercyler using the following cycle parameters: 1 cycle at 95 °C, 10 min; 95 °C, 15 s, 58–60 °C, 30–60 s; 72 °C, 30–60 s; 40 cycles of amplification. Ube3A mRNA was quantified and normalized to glyceraldehyde 3-phosphate dehydrogenase (GAPDH) and expressed as a fold induction compared to control conditions. Primer pairs for Ube3A: F_5′-CGAGGACAGATCACCAGGAG-3′ and R_5′-TCATTCGTGCAGGCCTCATT-3′.

### Rho-GTPase activity assay

Rho-GTPase pull-down assays were performed following the manufacturer’s protocol (Cytoskeleton, Inc.). Briefly, cells were washed once with ice-cold PBS and lysed with RIPA/M-PER buffer (1:1; ThermoFisher Scientific). Lysates were incubated on ice for 5 min, and centrifuged (12,000 × *g*) for 5 min. Then, 30 μl was used for input (total RhoA) and the remaining lysates were incubated with Rhotekin-RBD (RhoA) protein beads (25 μl) by rotating for 1 h at 4 °C. Beads were washed 3 times with ice-cold PBS (500 μl) and collected, and bound proteins (active RhoA)  were eluted with 1× sample buffer (30 μl). Input and eluted samples were then analyzed by gel electrophoresis and western blotting using either a RhoA-specific antibody.

### Statistics

All statistical analyses were performed using GraphPad Prism software. Statistical significance was determined by Student's *t*-test or one- or two-way analysis of variance (ANOVA), where appropriate, with a significance threshold of *p* < 0.05.

### Reporting summary

Further information on experimental design is available in the Nature Research Reporting Summary linked to this article.

## Supplementary information

Description of Supplementary Data

Supplementary Information

Supplementary Data 1

Reporting Summary

## Data Availability

The data supporting the findings of this study are available as Supplementary Data 1 and from the corresponding author upon request.
