# Supplementary material for: Dysfunction of the ubiquitin ligase E3A Ube3A/E6-AP contributes to synaptic pathology in Alzheimer’s disease
Source: Commun Biol. 2019 Mar 22;2:111. doi: 10.1038/s42003-019-0350-5 (PMC6430817; doi:10.1038/s42003-019-0350-5)
Supplement: Supplementary file 1 — Description of Supplementary Data [file 42003_2019_350_MOESM1_ESM.docx]

**Description of Additional Supplementary Files**

**File Name**: Supplementary Data 1

**Description**: Processed data. The graphs and statistical analyses were generated using the processed data presented in this table.
