# Supplementary material for: Dysfunction of the ubiquitin ligase E3A Ube3A/E6-AP contributes to synaptic pathology in Alzheimer’s disease
Source: Commun Biol. 2019 Mar 22;2:111. doi: 10.1038/s42003-019-0350-5 (PMC6430817; doi:10.1038/s42003-019-0350-5)
Supplement: Supplementary file 2 — Supplementary Information [file 42003_2019_350_MOESM2_ESM.pdf]

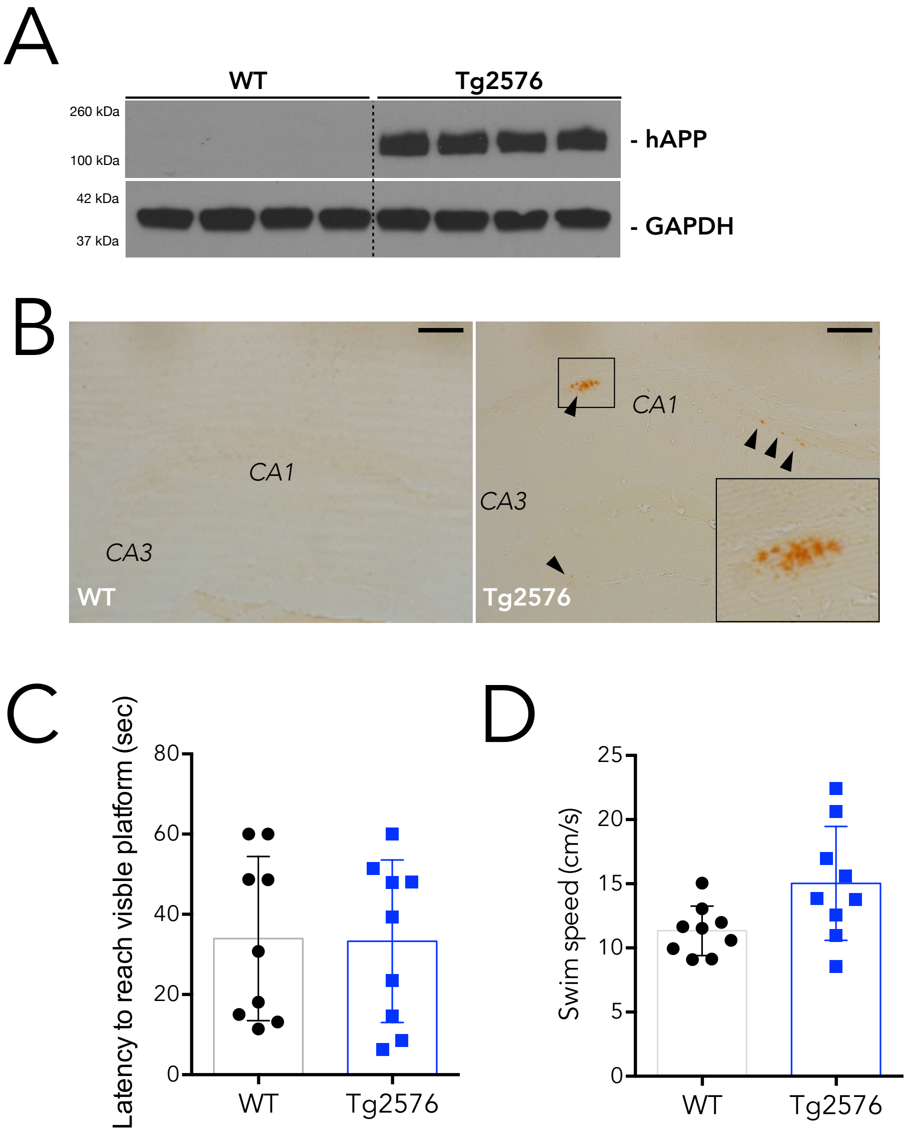


**Supplementary Figure 1. Tg2576 mice recapitulate features of AD pathology. (A)** Representative Western blot for hAPP and GAPDH (as house-keeping protein) of wild type (WT) and Tg2576 hippocampal lysates. **(B)** Brightfield images of Congo Red staining of wild type (WT) and Tg2576 hippocampus. Arrow heads indicate positive Aβ plaque staining. Inset shows a higher magnification view of a Congo red positive plaque. Scale bar = 200 μM. **(C)** Time required to reach the visible platform and **(D)** the swim speed for wild type (WT) and Tg2576 mice. Data presented as mean values +/- s.d. of N=6 WT and N=6 Tg2576. Note: Statistical analyses were performed using unpaired student's t-test for all experiments.

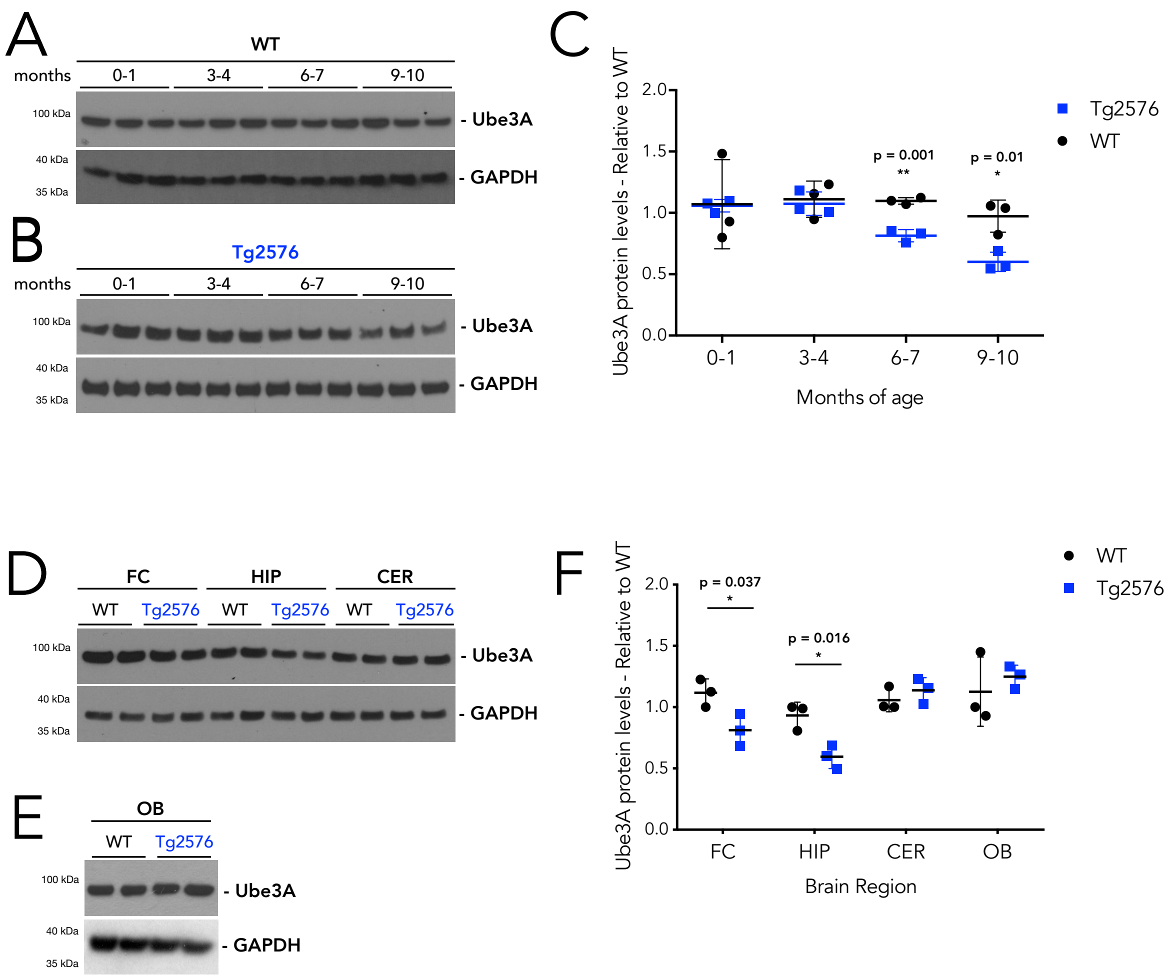


**Supplementary Figure 2. Age- and region-dependent decrease of Ube3A protein in Tg2576 mice. (A,B,D,E)** Representative Western blot showing levels Ube3A and in WT and Tg2576 mice at various ages in the hippocampus (A,B) and in various brain regions at 6-7 months of age (D,E). **(C,F)** Quantification of Western blot data showing decreased Ube3A protein levels in Tg2576 at 6-7 and 9-10 months of age relative to littermate WT mice, and decreased Ube3A protein levels in hippocampus (HIP) and frontal cortex (FC) of Tg2576 mice relative to littermate WT mice. Quantifications were normalized to GAPDH, as house-keeping gene. Data presented as mean +/- s.d. of N=3 WT and N=3 Tg2576 mice. Note: Statistical analyses were performed using two-way ANOVA followed by Bonferroni Post Hoc multiple comparisons (E), and multiple unpaired student's t-test for each brain region (H).

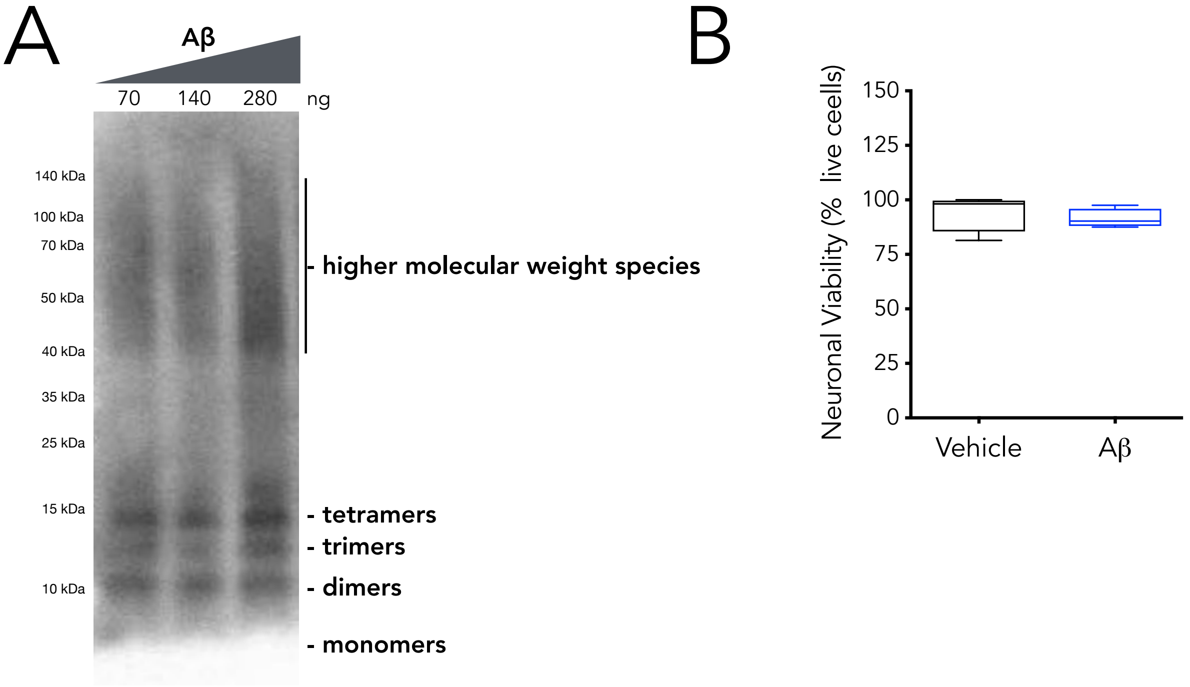


**Supplementary Figure 3. Characterization of oligomeric synthetic oAβ.** (**A**) Preparation of oligomeric Aβ results in the formation of various species, including dimers, trimers and tetramers, as well as various larger aggregated forms. (**B**) Survival data showing viability of neurons treated with 1.0 μM of oligomeric Aβ from (A) for 24 hours. Data presented as mean +/- Min. to Max values of N=4 independent cultures. Note: Statistical analyses were performed using unpaired student's t-test.

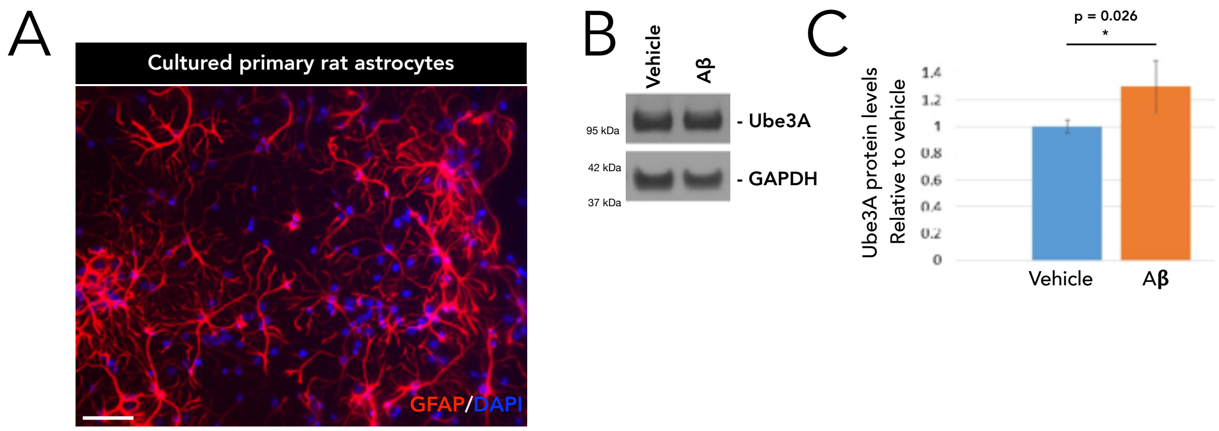


**Supplementary Figure 4. oAβ increases levels of Ube3A on primary rat astrocytes. (A)** Image of a representative field of primary rat astrocytes. (B-C) Representative Western blot (B) and quantification (C) of Ube3A of astrocytes treated with 1μM oAβ or Vehicle. Scale bar = 500 μM. Data presented as mean values +/- s.d. of N=3 independent cultures. Note: Statistical analyses were performed using unpaired student's t-test for all experiments.

A

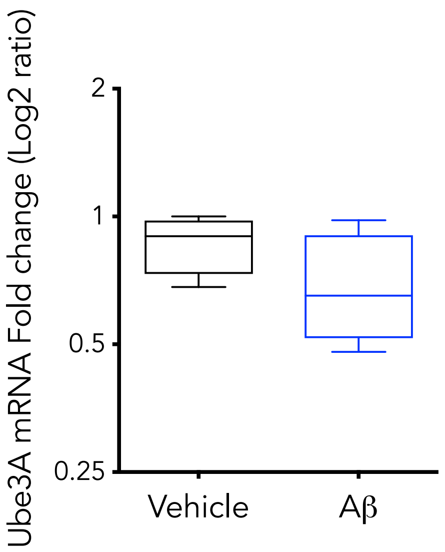


B

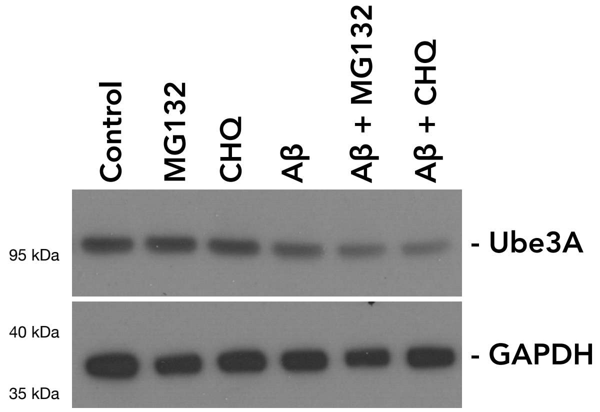


**Supplementary Figure 5. oAβ does not affect the transcription of Ube3A.** Quantitative-PCR analysis of Ube3A mRNA of hippocampal neurons treated with 1μM of oAβ (24h) or Vehicle. Note: Data presented as mean +/- Min. to Max values of N=4 independent cultures. Note: Statistical analyses were performed using unpaired student's t-test for all experiments.

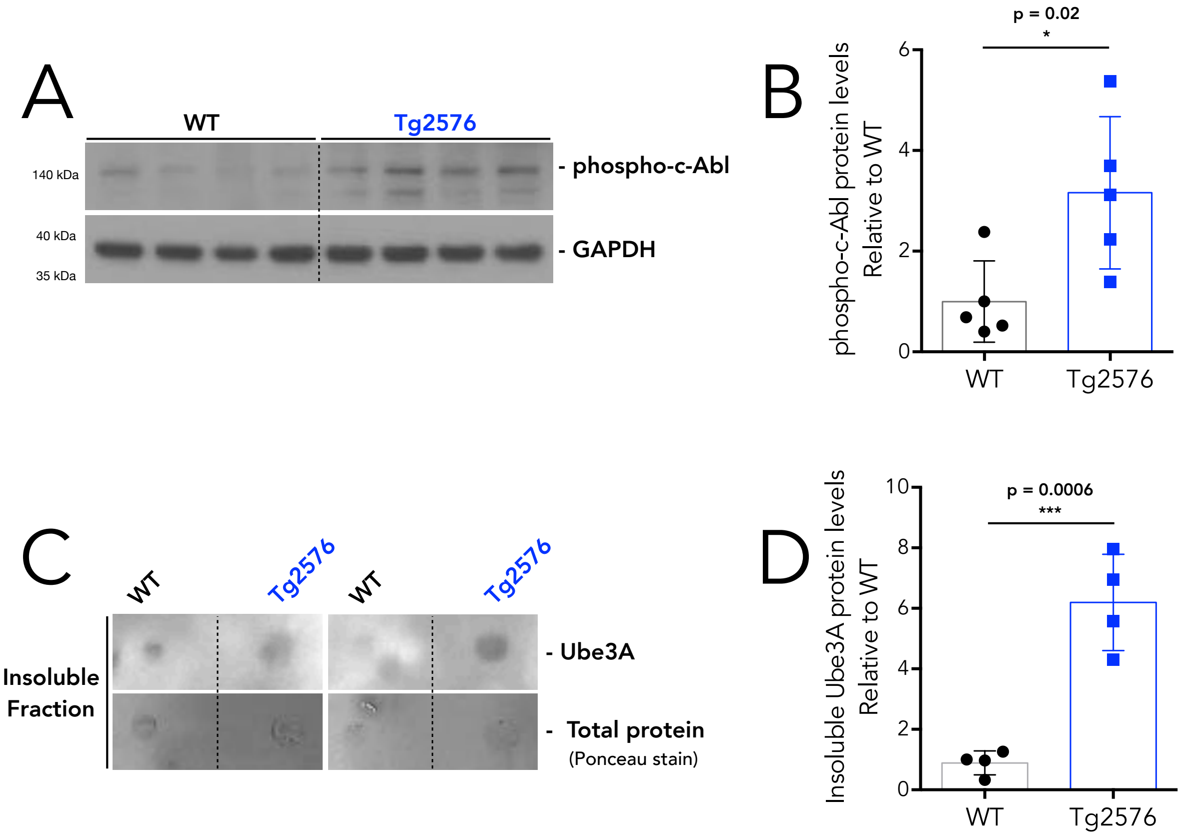


**Supplementary Figure 6. Phospho-c-Abl and insoluble Ube3A are increased in Tg2576 mouse model of AD. (A,C)** Representative Western blots showing levels of phospho-c-Abl in hippocampi of WT and Tg2576 mice, and levels of Ube3A in insoluble preparations from WT and Tg2576 brains. **(B,D)** Quantification of Western blot data showing increase phospho-c-Abl levels in Tg2576 hippocampi relative to WT mice, and increased insoluble Ube3A in Tg2576 brains relative to WT mice. Data presented as mean values +/- s.d. of N=4 WT and N=4 Tg2576 mice. Note: Statistical analyses were performed using unpaired student's t-test for all experiments.

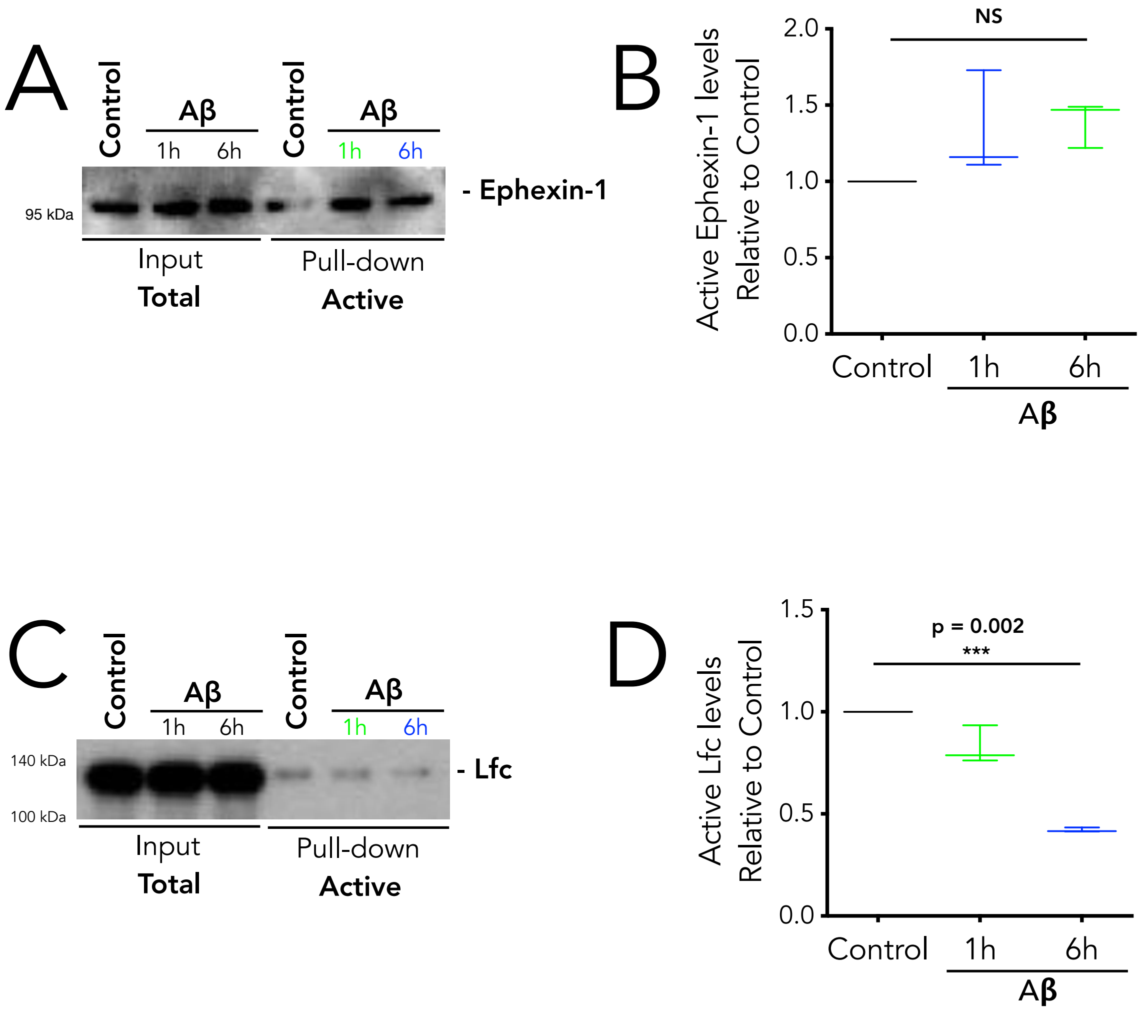


**Supplementary Figure 7. Aβ oligomers do not induce activation of other RhoA-specific GEFs, Ephexin-1 and Lfc. (A-B)** Representative Western blots showing levels of total and active Ephexin-1 and Lfc in vehicle- and oAβ-treated neuronal cultures. **(B,D)** Quantification of Western blot data showing no change in active Ephexin-1 (B) and decreased Lfc activity (D) in oAβ-treated (1h, 6h) cultures. Data presented as mean +/- Min. to Max values of N=3 independent cultures. Note: Statistical analyses were performed using one-way ANOVA followed by Bonferroni Post Hoc multiple comparisons for all experiments.

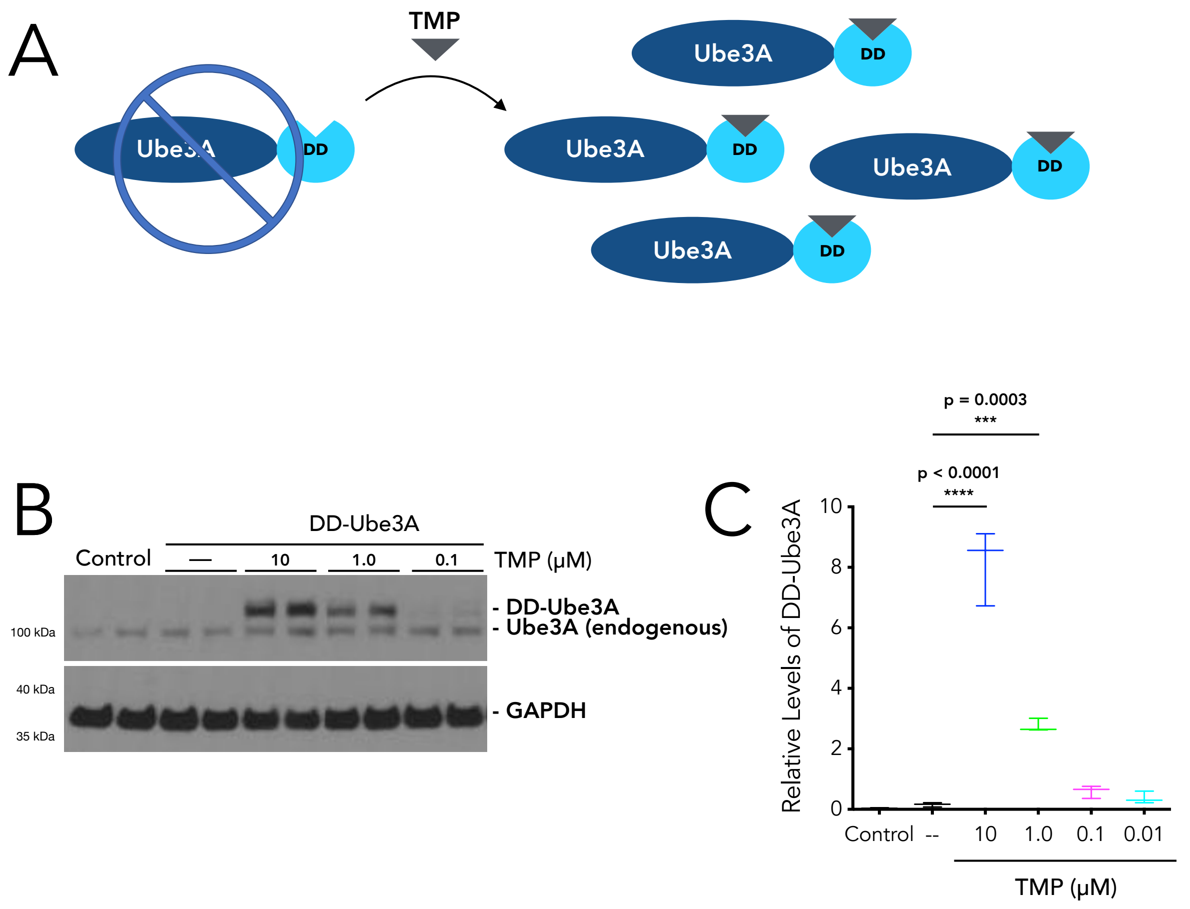


**Supplementary Figure 8. TMP treatment induces a dose-dependent accumulation of DD-Ube3A. (A)** Schematic diagram showing the mechanism DD destabilizing domain (DD)/TMP system. In absence of TMP, DD-conjugated Ube3a degrades after translation. TMP stabilizes the DD, and therefore expressed Ube3a stays available. **(B)** Representative Western blot (B) and quantification (C) of DD-Ube3a and GAPDH (as house-keeping protein) in rat hippocampal neurons. Data presented as mean +/- Min. to Max. values of N=3 independent cultures. Note: Statistical analyses were performed using one-way ANOVA followed by Tukey Post Hoc multiple comparisons for all experiments.

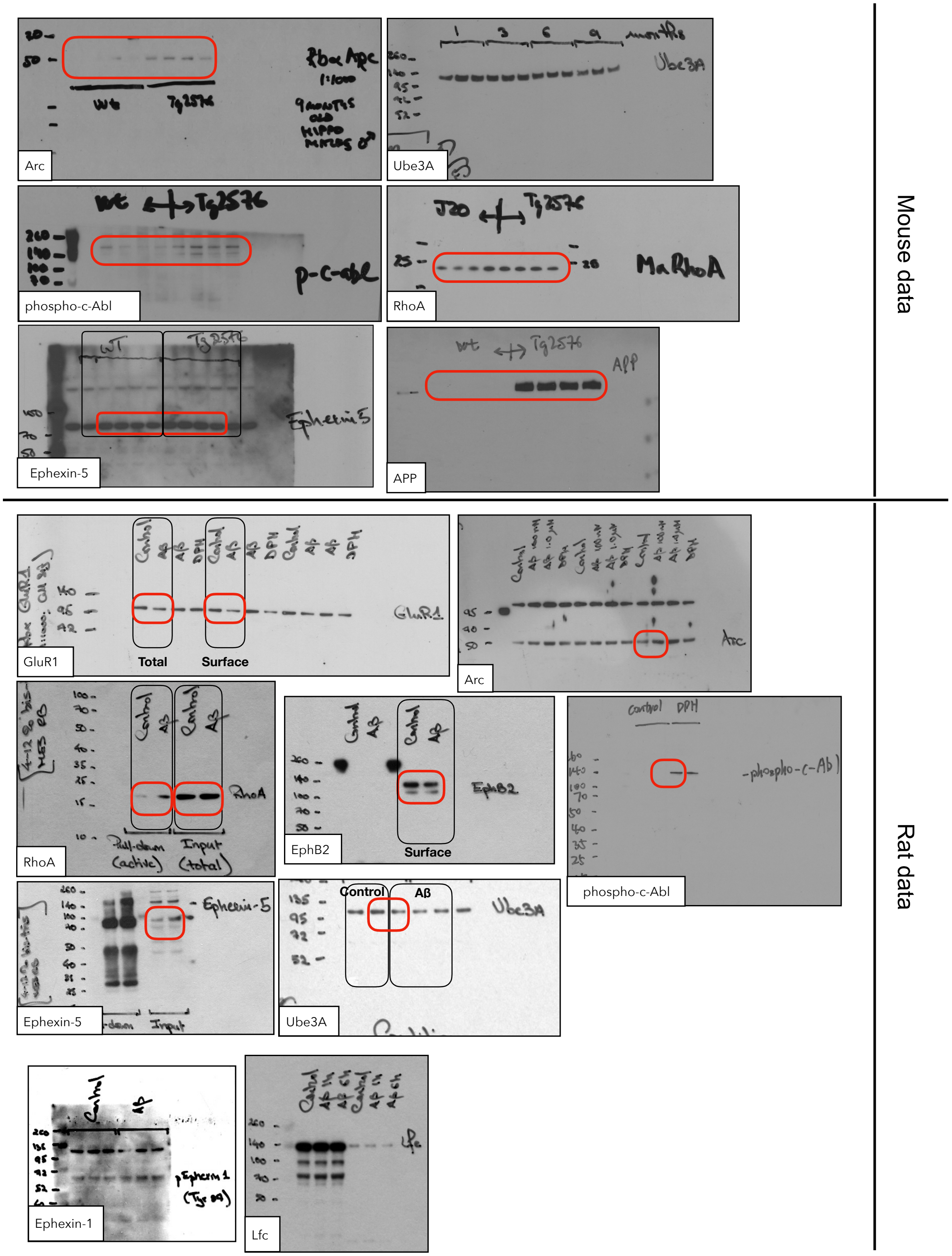


**Supplementary Figure 9: Full Western blot images.** Representative Western blot images used in these studies.

| Primary Antibodies | Host | Company | Cat. Number | Application | Supp. Ref. |
|---|---|---|---|---|---|
| Arc | Rabbit | Santa Cruz | sc-15325 | WB | 8, 9 |
| Aβ | Mouse | Covance | 803001 | WB | |
| EphB2 | Rabbit | Millipore | MABN726 | WB | |
| Ephexin-1 | Rabbit | ECM | EP2821 | WB | |
| Ephexin-5 | Rabbit | Novus | NBP2-15455 | WB | |
| GAPDH | Rabbit | Cell Signaling | 2118 | WB | |
| GluR1 | Mouse | Millipore | MAB2263 | WB, IF | 6, 7 |
| HA | Rabbit | Cell Signaling | 2367 | WB, IP | |
| hAPP | Mouse | ThermoFisher Scientific | 14-9749-80 | WB, IHC | |
| Lfc | Rabbit | Cell Signaling | #4076 | WB | 13 |
| p53 | Mouse | Santa Cruz | sc-126 | WB | |
| phospho-c-Abl (Tyr412) | Rabbit | Cell Signaling | #2865 | WB | 12 |
| phospho-Tyrosine | Mouse | Cell Signaling | #9411 | WB | |
| PSD95 | Rabbit | Cell Signaling | #2507 | WB | |
| RhoA | Rabbit | Santa Cruz | sc-418 | WB | 4, 5 |
| RhoA | Mouse | Cell Signaling | #2117 | WB | 10, 11 |
| Ube3A | Rabbit | Cell Signaling | #7526 | WB | 1, 2, 3 |
| βIII-tubulin | Rabbit | Abcam | ab18207 | IF | |
| | | | | | |
| **Secondary Antibodies** | | **Company** | **Cat. Number** | **Application** | |
| Anti-mouse HRP | Goat | Cell Signaling | 7076 | WB | |
| Anti-rabbit HRP | Goat | Cell Signaling | 7074 | WB | |
| anti-mouse Alexa 568 | Goat | ThermoFisher Scientific | A-11004 | IF | |
| anti-mouse Alexa 488 | Goat | ThermoFisher Scientific | A-11001 | IF | |

**Supplementary Table 1: Antibodies used in these studies.**

**Supplementary References**


1. Saez, I., Koyuncu, S., Gutierrez-Garcia, R., Dieterich, C. & Vilchez, D. Insights into the ubiquitin-proteasome system of human embryonic stem cells. *Sci Rep* **8**, 4092, doi:10.1038/s41598-018-22384-9 (2018).
2. Pulimood, N. S., Rodrigues, W. D. S. J., Atkinson, D. A., Mooney, S. M. & Medina, A. E. The Role of CREB, SRF, and MEF2 in Activity-Dependent Neuronal Plasticity in the Visual Cortex. *J Neurosci* **37**, 6628-6637, doi:10.1523/JNEUROSCI.0766-17.2017 (2017).
3. Krishnan, V. *et al.* Autism gene Ube3a and seizures impair sociability by repressing VTA Cbln1. *Nature* **543**, 507-512, doi:10.1038/nature21678 (2017).
4. Hottman, D. *et al.* Systemic or Forebrain Neuron-Specific Deficiency of Geranylgeranyltransferase-1 Impairs Synaptic Plasticity and Reduces Dendritic Spine Density. *Neuroscience* **373**, 207-217, doi:10.1016/j.neuroscience.2018.01.026 (2018).
5. Lian, G., Chenn, A., Ekuta, V., Kanaujia, S. & Sheen, V. Formin 2 Regulates Lysosomal Degradation of Wnt-Associated beta-Catenin in Neural Progenitors. *Cereb Cortex*, doi:10.1093/cercor/bhy073 (2018).
6. Mignogna, M. L. *et al.* The intellectual disability protein RAB39B selectively regulates GluA2 trafficking to determine synaptic AMPAR composition. *Nat Commun* **6**, 6504, doi:10.1038/ncomms7504 (2015).
7. Atkin, G. *et al.* Loss of F-box only protein 2 (Fbxo2) disrupts levels and localization of select NMDA receptor subunits, and promotes aberrant synaptic connectivity. *J Neurosci* **35**, 6165-6178, doi:10.1523/JNEUROSCI.3013-14.2015 (2015).
8. Soule, J. *et al.* Balancing Arc synthesis, mRNA decay, and proteasomal degradation: maximal protein expression triggered by rapid eye movement sleep-like bursts of muscarinic cholinergic receptor stimulation. *J Biol Chem* **287**, 22354-22366, doi:10.1074/jbc.M112.376491 (2012).
9. Cao, C. *et al.* Impairment of TrkB-PSD-95 signaling in Angelman syndrome. *PLoS Biol* **11**, e1001478, doi:10.1371/journal.pbio.1001478 (2013).
10. Zhang, Y. *et al.* Down-regulation of Skp2 expression inhibits invasion and lung metastasis in osteosarcoma. *Sci Rep* **8**, 14294, doi:10.1038/s41598-018-32428-9 (2018).
11. Zhang, Y. L. *et al.* SPON2 Promotes M1-like Macrophage Recruitment and Inhibits Hepatocellular Carcinoma Metastasis by Distinct Integrin-Rho GTPase-Hippo Pathways. *Cancer Res* **78**, 2305-2317, doi:10.1158/0008-5472.CAN-17-2867 (2018).
12. Nasr, R. R. *et al.* ST1926, an orally active synthetic retinoid, induces apoptosis in chronic myeloid leukemia cells and prolongs survival in a murine model. *Int J Cancer* **137**, 698-709, doi:10.1002/ijc.29407 (2015).
13. Park, J. *et al.* Mechanochemical feedback underlies coexistence of qualitatively distinct cell polarity patterns within diverse cell populations. *Proc Natl Acad Sci U S A* **114**, E5750-E5759, doi:10.1073/pnas.1700054114 (2017).
