# Supplementary material for: Dysfunction of the ubiquitin ligase E3A Ube3A/E6-AP contributes to synaptic pathology in Alzheimer’s disease
Source: Commun Biol. 2019 Mar 22;2:111. doi: 10.1038/s42003-019-0350-5 (PMC6430817; doi:10.1038/s42003-019-0350-5)
Supplement: Supplementary file 4 — Reporting Summary [file 42003_2019_350_MOESM4_ESM.pdf]

nature research

Corresponding author(s): Roger Lefort

Last updated by author(s): Dec 20, 2018

Reporting Summary

Nature Research wishes to improve the reproducibility of the work that we publish. This form provides structure for consistency and transparency in reporting. For further information on Nature Research policies, see Authors & Referees and the Editorial Policy Checklist.

Statistics

For all statistical analyses, confirm that the following items are present in the figure legend, table legend, main text, or Methods section.

n/a	Confirmed	
☐	☒	The exact sample size (*n*) for each experimental group/condition, given as a discrete number and unit of measurement
☐	☒	A statement on whether measurements were taken from distinct samples or whether the same sample was measured repeatedly
☐	☒	The statistical test(s) used AND whether they are one- or two-sided<br>*Only common tests should be described solely by name; describe more complex techniques in the Methods section.*
☒	☐	A description of all covariates tested
☒	☐	A description of any assumptions or corrections, such as tests of normality and adjustment for multiple comparisons
☐	☒	A full description of the statistical parameters including central tendency (e.g. means) or other basic estimates (e.g. regression coefficient) AND variation (e.g. standard deviation) or associated estimates of uncertainty (e.g. confidence intervals)
☐	☒	For null hypothesis testing, the test statistic (e.g. F, t, r) with confidence intervals, effect sizes, degrees of freedom and P value noted<br>*Give P values as exact values whenever suitable.*
☒	☐	For Bayesian analysis, information on the choice of priors and Markov chain Monte Carlo settings
☒	☐	For hierarchical and complex designs, identification of the appropriate level for tests and full reporting of outcomes
☒	☐	Estimates of effect sizes (e.g. Cohen's *d*, Pearson's *r*), indicating how they were calculated

Our web collection on statistics for biologists contains articles on many of the points above.

Software and code

Policy information about availability of computer code

Data collection	Densitometric analysis of Western blot data and microscopy analysis were performed using the open source software Image J (https://imagej.nih.gov/ij/) and NeuronStudio (http://research.mssm.edu/cnic/tools-ns.html).
Data analysis	All statistical analyses were performed using GraphPad Prism software v7 and v8.

For manuscripts utilizing custom algorithms or software that are central to the research but not yet described in published literature, software must be made available to editors/reviewers. We strongly encourage code deposition in a community repository (e.g. GitHub). See the Nature Research guidelines for submitting code & software for further information.

Data

Policy information about availability of data

All manuscripts must include a data availability statement. This statement should provide the following information, where applicable:

- Accession codes, unique identifiers, or web links for publicly available datasets
- A list of figures that have associated raw data
- A description of any restrictions on data availability

The data supporting the findings of this study are available as supplementary data and from the corresponding author upon request.

Field-specific reporting

Please select the one below that is the best fit for your research. If you are not sure, read the appropriate sections before making your selection.

☒ Life sciences ☐ Behavioural & social sciences ☐ Ecological, evolutionary & environmental sciences

Life sciences study design

All studies must disclose on these points even when the disclosure is negative.

Sample size	For behavioral experiments, we expected that a minimum of 10 animals will be required for each group. An N of 10 per group provides a power of 90% for detecting a difference at alpha = 0.05 with an effect size f=0.5. (calculated using G*Power: http://www.psycho.uni-duesseldorf.de/aap/projects/gpower/).
Data exclusions	1 data point in the probe test was removed from the Tg2576 group due the death on the one of the animal prior to open platform test. Removal of data point did not alter the results.
Replication	All in vitro experiments were performed in at least dupliucates, and repeated independently from at least 3 cultures.
Randomization	Mice (WT and Tg2576) were coded and randomly divided into 2 groups for training and probe test.
Blinding	Data acquisition and analysis for behavioral experiments were performed blinded, with the experimenter not knowing the genotype of the animals. Data analysis for microscopy studies were performed blinded with each culture coded.

Reporting for specific materials, systems and methods

We require information from authors about some types of materials, experimental systems and methods used in many studies. Here, indicate whether each material, system or method listed is relevant to your study. If you are not sure if a list item applies to your research, read the appropriate section before selecting a response.

Materials & experimental systems

n/a	Involved in the study	
☐	☒	Antibodies
☐	☒	Eukaryotic cell lines
☒	☐	Palaeontology
☐	☒	Animals and other organisms
☒	☐	Human research participants
☒	☐	Clinical data

Methods

n/a	Involved in the study	
☒	☐	ChIP-seq
☒	☐	Flow cytometry
☒	☐	MRI-based neuroimaging

Antibodies

Antibodies used

Western blot:
UBE3A (D10D3) Rabbit mAb, Cell Signaling #7526, Lot 1
RhoA (67B9) Rabbit mAb, Cell Signaling #2117, Lot 3
Rho A (26C4) Mouse Ab, Santa Cruz #sc-418, Lots C1011 and D0615
Phospho-c-Abl (Tyr412) (247C7) Rabbit mAb, Cell Signaling #2865, Lot 3
GAPDH (D16H11) XP® Rabbit mAb, Cell Signaling #5174, Lot 10
HA-Tag (C29F4) Rabbit mAb, Cell Signaling #3724, Lot 8
PSD95 (D27E11) XP® Rabbit mAb, Cell Signaling #3450, Lot 1
p53 (DO-1) mouse Ab, Santa Cruz sc-126, Lot A2413
Arc (H300) mouse Antibody (40): sc-15325, Lot B0615 (discontinued)
EphB2 Rabbit Ab, Millipore #AB15076, Lot 1970338
Ephexin-5 Rabbit Ab, Novus #NBP2-15455 Lot 40646 and Abcam #ab127704 Lot GR105451-1
APP (22C11) mouse Ab, Millipore #MAB348 Lot 2106931
AMPA Receptor 1 (GluA1) (D4N9V) Rabbit mAb, Cell Signaling #13185, Lot 1
GEF-H1 (Lfc) Rabbit Ab, Cell Signaling #4145, Lot 1 (discontinued)
Ephexin-1 Rabbit Ab, ECM Biosciences #EP2821,

Immunocytochemistry
AMPA GluR1 (RH95) Mouse mAb, Millipore, #04-855

Validation

- UBE3A (D10D3) Rabbit mAb, Cell Signaling #7526, Lot 1 for WB
Validated through shRNA depletion in cell lines; other publications, Nature Communications on 23 July 2018 by Koyuncu, S., Saez, I., et al., Scientific Reports on 6 March 2018 by Saez, I., Koyuncu, S., et al., The Journal of Neuroscience on 12 July 2017 by Pulimood, N. S., Rodrigues, W. D. S., et al., Nature on 23 March 2017 by Krishnan, V., Stoppel, D. C., et al..

- RhoA (67B9) Rabbit mAb, Cell Signaling #2117, Lot 3 and Rho A (26C4) Mouse Ab, Santa Cruz #sc-418, Lots C1011 and D0615

for WB
Validated through positive-control experiments with RhoA activators and through plasmid-mediated expression.

- Phospho-c-Abl (Tyr412) (247C7) Rabbit mAb, Cell Signaling #2865, Lot 3 for WB
Validated through c-Abl activator experiments with DPH
Other publications, Cell Research on 1 November 2018 by Yan, F., Al-Kali, A., et al., Cell Chemical Biology on 19 October 2017 by Diaz, J. E., Morgan, C. W., et al., Scientific Reports on 18 September 2017 by Wang, Y., Hall, R. A., et al., Nature Communications on 17 November 2014 by Lamontanara, A. J., Georgeon, S., et al..

- p53 (DO-1) mouse Ab, Santa Cruz sc-126, Lot A2413 for WB
Other publications, Xue, YN. et al. 2019. Exp. Cell Res. 374: 249-258, Lee, Y. et al. 2018. Nat Commun. 9: 2301, Adamson, SE. et al. 2018. Arterioscler. Thromb. Vasc. Biol. 38: 1020-1029, Li, X. et al. 2018. Cell Death Differ.

- Arc (H300) mouse Antibody (40): sc-15325, Lot B0615 for WB
Other publications, Bojovic, O. et al. 2015. PloS one. 10: e0123604, Cao, C. et al. 2013. PLoS biology. 11: e1001478, O'Keeffe, SM. et al. 2012. Neuroscience. 201: 219-230, Soulé, J. et al. 2012. The Journal of biological chemistry. 287: 22354-66.

- EphB2 Rabbit Ab, Millipore #AB15076, Lot 1970338 for WB
Validated through shRNA depletion in cell lines

- APP (22C11) mouse Ab, Millipore #MAB348 Lot 2106931 for WB
Validated in B103 cells which do not express APP

- Ephexin-5 Rabbit Ab, Novus #NBP2-15455 Lot 40646 and Abcam #ab127704 Lot GR105451-1 for WB
Validated through siRNA depletion in neurons and cell lines

- AMPA Receptor 1 (GluA1) (D4N9V) Rabbit mAb, Cell Signaling #13185, Lot 1 for WB
Validated though shRNA depletion in cell lines

- GEF-H1 (Lfc) Rabbit Ab, Cell Signaling #4145, Lot 1 (discontinued) for WB
Other publications, Proceedings of the National Academy of Sciences of the United States of America on 11 July 2017 by Park, J., Holmes, W. R., et al., Nature Communications on 26 June 2017 by Takano, T., Wu, M., et al., Journal of Cell Science on 1 March 2017 by Jiu, Y., Peränen, J., et al., Molecular Biology of the Cell on 1 May 2016 by Scott, D. W., Tolbert, C. E., et al..

- Ephexin-1 Rabbit Ab, ECM Biosciences #EP2821 for WB
Other publications, Rosas, O.R. (2014) Neural regeneration research 9(24): 2164. WB: rat muscle, Rosas, O.R. (2014) Neural regeneration research 9(24): 2164. WB: rat muscle, Rosas, O.R. et al. (2011) Dev Neurobiol. 71(7):595. WB, IHC: rat spinal cord.

- PSD95 (D27E11) XP® Rabbit mAb, Cell Signaling #3450, Lot 1 for WB
Other publications, Cell Death & Disease on 6 November 2018 by Aubrecht, T. G., Faden, A. I., et al., Nature Communications on 22 October 2018 by Zhao, N., Liu, C. C., et al., The EMBO Journal on 27 June 2018 by Truckenbrodt, S., Viplav, A., et al., Scientific Reports on 14 March 2018 by Pitts, E. G., Li, D. C., et al..

- AMPA GluR1 (RH95) Mouse mAb, Millipore, #04-855 for ICC
Validated though shRNA depletion in cell lines

Eukaryotic cell lines

Policy information about cell lines

Cell line source(s)	B35 (ATCC® CRL-2754™) cell line was used in these studies.
Authentication	B35 cell line was not tested for mycoplasma contamination
Mycoplasma contamination	Cell lines were not tested for mycoplasma contamination
Commonly misidentified lines (See ICLAC register)	n/a

Animals and other organisms

Policy information about studies involving animals; ARRIVE guidelines recommended for reporting animal research

Laboratory animals	For these studies we used the Tg2576 transgenic mouse line carrying a transgene coding for the 695-amino acid isoform of human Alzheimer β-amyloid (Aβ) precursor protein carrying the Swedish mutation on a C57BL/6 background. C57BL/6 wild-type mice were used as control animals.

Wild animals	n/a
Field-collected samples	n/a
Ethics oversight	All experiments involving animals were performed under the guidelines of the Columbia University Institutional Animal Care and Use Committee (IACUC).

Note that full information on the approval of the study protocol must also be provided in the manuscript.
